# A WD40 Protein Encoding Gene *Fvcpc2* Positively Regulates Mushroom Development and Yield in *Flammulina velutipes*

**DOI:** 10.3389/fmicb.2020.00498

**Published:** 2020-03-26

**Authors:** Taju Wu, Zhenying Zhang, Chengcheng Hu, Long Zhang, Shenglong Wei, Shaojie Li

**Affiliations:** ^1^State Key Laboratory of Mycology, Institute of Microbiology, Chinese Academy of Sciences, Beijing, China; ^2^College of Life Sciences, University of Chinese Academy of Sciences, Beijing, China; ^3^Shandong Jinniu Biotech Company Limited, Jinan, China; ^4^Gansu Engineering Laboratory of Applied Mycology, Hexi University, Zhangye, China

**Keywords:** *Flammulina velutipes*, WD40 protein, sexual development, mushroom breeding, *Neurospora crassa*

## Abstract

Ascomycota and Basidiomycota are two closely related phyla and fungi in two phyla share some common morphological developmental process during fruiting body formation. In *Neurospora crassa*, the Gβ-like protein CPC-2 with a seven-WD40 repeat domain was previously reported. By transforming CPC-2 ortholog encoding genes, from 7 different fungal species across Ascomycota and Basidiomycota, into the *cpc-2* deletion mutant of *N. crassa*, we demonstrate that all tested CPC-2 ortholog genes were able to complement the defects of the *cpc-2* deletion mutant in sexual development, indicating that CPC-2 proteins from Ascomycota and Basidiomycota have the similar cellular function. Using *Flammulina velutipes* as a model system for mushroom species, the CPC-2 ortholog FvCPC2 was characterized. *Fvcpc2* increased transcription during fruiting body development. Knockdown of *Fvcpc2* by RNAi completely impaired fruiting body formation. In three *Fvcpc2* knockdown mutants, transcriptional levels of genes encoding adenylate cyclase and protein kinase A catalytic subunit were significantly lower and colony growth became slower than wild type. The addition of cAMP or the PKA-activator 8-Bromo-cAMP into the medium restored the *Fvcpc2* knockdown mutants to the wild-type colony growth phenotype, suggesting that the involvement of cAMP production in the regulatory mechanisms of FvCPC2. Knockdown of *Fvcpc2* also weakened transcriptional responses to sexual development induction by some genes related to fruiting body development, including 4 jacalin-related lectin encoding genes, 4 hydrophobin encoding genes, and 3 functionally-unknown genes, suggesting the participation of these genes in the mechanisms by which FvCPC2 regulates fruiting body development. All three *Fvcpc2* overexpression strains displayed increased mushroom yield and shortened cultivation time compared to wild type, suggesting that *Fvcpc2* can be a promising reference gene for Winter Mushroom breeding. Since the orthologs of FvCPC2 were highly conserved and specifically expressed during fruiting body development in different edible mushrooms, genes encoding FvCPC2 orthologs in other mushroom species also have potential application in breeding.

## Introduction

Among the six phyla of the fungal kingdom, Ascomycota members (produce sexual spores enclosed in a special sac called an ascus) and Basidiomycota members (produce external basidiospores on a basidium during sexual life) are closely related and together form the fungal subkingdom of Dikarya (Hibbett et al., 2007; Kirk et al., 2008). They evolved into two different branches only around 500 million years ago (Berbee and Taylor, 2010; Oberwinkler, 2012). The two phyla share commonness in the morphological process during fruiting body development (Pelkmans et al., 2016).

Fungal fruiting is an elaborate process which is corporately controlled by both genetics and environmental factors (Kües, 2000). As previously reported, common regulators of fruiting body development exist between these two phyla. For instance, the light perception mechanism plays a pivotal role in light-dependent fruiting. The blue light receptor WC-1 regulates the formation of perithecia (fruiting bodies) in the ascomycetes *Neurospora crassa* and *Trichoderma reesei* (Chen et al., 2012; Linden et al., 1997). Its orthologs in the basidiomycete mushroom *Schizophyllum commune* and *Coprinopsis cinerea* are required for the initiation and maturation of basidioma, respectively (Terashima et al., 2005; Ohm et al., 2013). Another common regulatory system of fruiting between these two phyla is pheromone signaling. Pheromone reception and its signal transduction are essential for mating and for fruiting initiation in many species of these two phyla (Debuchy et al., 2010; Raudaskoski and Kothe, 2010). The membrane-located heterotrimeric G-protein complex participates in pheromone signaling in many fungal species (Lengeler et al., 2000; Brown and Casselton, 2001; Li et al., 2007). The Gβ subunit of heterotrimeric G-proteins belong to WD40 repeat protein family, in which each of WD40 domains folds into a β-propeller structure, providing a platform for protein-protein interaction (Jain and Pandey, 2018; Neer et al., 1994). In addition to Gβ subunit, WD40 repeat proteins also have many other members.

In *N. crassa*, a Gβ-like protein CPC-2 with a seven-WD40 repeat domain controls amino acid synthesizing enzymes and protoperithecium development (Krüger et al., 1990; Müller et al., 1995). Several CPC-2 orthologs in other fungal species of the Ascomycota and Basidiomycota phyla, including Cpc2 of *Schizosaccharomyces pombe* (Won et al., 2001), Asc1 of *Candida albicans* (Liu et al., 2010), CpcB of *Aspergillus nidulans* (Kong et al., 2013), Gib2 of *Cryptococcus neoformans* (Palmer et al., 2006), and Rak1 of *Ustilago maydis* (Wang et al., 2011), were also reported. Cpc2 regulates the cell cycle and sexual differentiation in *S. pombe* (McLeod et al., 2000; Jeong et al., 2004; Paul et al., 2009), CpcB participates in the hyphal growth and cleistothecium development in *A. nidulans* (Hoffmann et al., 2000; Kong et al., 2013) and Rak1 controls mating in *U. maydis* (Wang et al., 2011). These studies indicate that CPC-2 and its orthologs are involved in fruiting in different fungal species in both Ascomycota and Basidiomycota. However, it is unknown whether they have the same cellular function. Although protoperithecium and cleistothecium of Ascomycota fungi are dramatically different from basidioma of Basidiomycota fungi in structure, they are all specialized fruiting body structures with similar biological functions. CPC-2 and CpcB regulate fruiting body development in *N. crassa* and *A. nidulans*, respectively (Müller et al., 1995; Kong et al., 2013). Therefore, it is possible that their orthologs in Basidiomycota fungi regulates mushroom development.

Although orthologs of CPC-2 have been predicted in the genomes of several mushrooms, including model mushroom species (*C. cinerea* and *S. commune*) and cultivated mushrooms (*Flammulina velutipes*, *Volvariella volvacea*, *Pleurotus ostreatus*, *Ganoderma lucidum*, and *Lentinus edodes*) (Ohm et al., 2010; Stajich et al., 2010; Chen et al., 2012; Grigoriev et al., 2012; Bao et al., 2013; Park et al., 2014; Riley et al., 2014; Sakamoto et al., 2017), none of them has been functionally characterized. Similarly as that *cpc-2* is up-regulated after crossing in *N. crassa* (Lehr et al., 2014), CPC-2 orthologs have an increased expression during fruiting body development in many mushroom species (Ohm et al., 2010; Yu et al., 2012; Cheng et al., 2013; Liu et al., 2015; Zhang et al., 2015; Sakamoto et al., 2017), suggesting that CPC-2 orthologs are likely involved in fruiting body development in these mushroom species. If it is true, CPC-2 ortholog encoding genes have potential to be a new group of reference genes for mushroom breeding. To date, the number of reported genes involved in mushroom development is very limited and most of them were only studied in model mushrooms (*C. cinerea* or *S. commune*). Identification of new genes critical for fruiting body development in commercially cultivated mushroom is necessary.

Due to the available genetic techniques (Kim et al., 2010; Okamoto et al., 2010; Hatoh et al., 2013; Shi et al., 2016), the world-widely cultivated Winter Mushroom (*F. velutipes*, which was recently renamed as *Flammulina filiformis*; Wang et al., 2018) has been used as a model to study mushroom development (Park et al., 2014; Lu et al., 2016). In this study, we intended to test whether CPC-2 orthologs are functionally conserved for fruiting body development in the two phyla Ascomycota and Basidiomycota. We also aimed to analyze the role of CPC-2 orthologs in fruiting body development in mushroom species using *F. velutipes* as a model system.

## Materials and Methods

### Strains and Media

The dikaryotic strain F19 and the monokaryotic strain L11 of *F. velutipes*, the cDNA of *V. volvacea*, *G. lucidum*, and *P. ostreatus* and the binary vector pBHg-BCA1 were provided by Professor Baogui Xie (Fujian Edible Fungi Germplasm Resource Collection Center of China) (Liu et al., 2015; Wang et al., 2015). The cDNA of *Saccharomyces cerevisiae*, *C. albicans*, and *C. neoformans* were obtained from Professor Fengyan Bai, Guanghua Huang, and Linqi Wang (Institute of Microbiology, Chinese Academy of Sciences), respectively.

CYM medium (w v^–1^, 0.2% yeast extract, 0.2% tryptone, 1% maltose, and 2% glucose) was used for routine growth of *F. velutipes* (Lin et al., 2013). For fruiting body development, *F. velutipes* strains were cultured in the composted sawdust substrate (52.5% cottonseed hulls, 25% wheat bran, 15% sawdust, 5% corn flour, 2% gypsum, and 0.5% ground limestone) with 61% moisture content (Wang et al., 2015). *Escherichia coli* DH5α was used for propagation of plasmids, and *Agrobacterium tumefaciens* AGL-1 was used for transforming the plasmids into *F. velutipes*.

Strains FGSC#4200 (wild type, mating type a), FGSC#2225 (wild type, mating type A) and FGSC#13695 (the deletion mutant of *cpc-2*, mating type a, heterokaryon) of *N. crassa* were obtained from the Fungal Genetics Stock Center (FGSC^1^; University of Kansas Medical Center). These strains were routinely grown on Vogel’s minimal medium with 2% sucrose for conidial production or on synthetic crossing medium (SCM) with 0.2% sucrose for protoperithecium and perithecium formation (Davis and Serres, 1970). The 150 mm slants of solid agar medium (2% agar, 0.5% sucrose, 0.1 × SC and 1 mM iodoacetate) were used to separate the homokaryon (Ebbole and Sachs, 1990).

### Homokaryonization of the *cpc-2* Deletion Mutant

FGSC#13695 is a heterokaryon of the *cpc-2* deletion mutant which could produce normal protoperithecia as wild type. Therefore homokaryonization was performed to obtain the absolute homokaryons of microconidia (Ebbole and Sachs, 1990). Firstly, the mycelia plug (*d* = 5 mm) of FGSC#13695 was inoculated on slants and grown at 25°C with a 12 h light/dark cycle for 10 days to produce microconidia. Secondly, the microconidia were harvested from each slant into 2.5 ml of sterile water and filtered through a 5 μm PVDF filter units (Sterile Millex Filter Units; Merck KGaA, Darmstadt, Germany). Thirdly, the microconidia (2000 spores) were spotted on the Vogel’s/sorbose agar plate and incubated at 28°C for 2–3 days. Lastly, individual colonies were transferred to Vogel’s slants for confirming homokaryonization using PCR by three disparate primers (cpc2-ORF-F/R, cpc2-up-F/R, and cpc2-down-F/R) and fertility tests. The final sterile homokaryon strain was used as the Δ*cpc-2* strain in this study. The primers of homokaryonization were shown in Supplementary Table S1.

### Sequence Acquisition

The sequence of *Fvcpc2* (GenBank Accession No. KY815023) was acquired from the genome of *F. velutipes* strain L11. The sequence of *Vvcpc2* (GenBank Accession No. MN075138) was obtained from the genome of *V. volvacea* strain PYd21.

The sequence of CPC-2 orthologs encoding genes (with GenBank No. NM_001182616.1, XM_019475482.1, XP_012053792.1, GQ293361.1, and KDQ27929.1, respectively) from *S. cerevisiae*, *C. albicans*, *C. neoformans*, *G. lucidum*, and *P. ostreatus* were obtained from National Center for Biotechnology Information (NCBI)^2^.

### Total DNA Extraction

For DNA extraction of *F. velutipes*, the mycelia grown on cellophane-covered CYM agar at 25°C for 7 days were harvested and extracted by a modified CTAB (Cetyltrimethyl ammonium Bromide) approach (Porebski et al., 1997).

For DNA extraction of *N. crassa*, the mycelia were cultured in Vogel’s liquid medium (1 × Vogel’s salts with 2% glucose) at 28°C for 24 h and extracted by the ethanolic perchlorate method (Metzenberg and Baisch, 1981).

### Complementation of the *cpc-2* Deletion Mutant of *N. crassa*

The vector pCB1532, which contains a sulfonylurea resistance allele of the *Magnaporthe grisea ILV*1 as a selectable marker (Sweigard et al., 1997), was used in the construction. In order to achieve functional complementing plasmids, the coding sequences of *Fvcpc2*, *cpc2*, *asc1*, *Glcpc2*, *Vvcpc2*, *Pocpc2*, *gib2* of *cpc-2* orthologs from *F. velutipes*, *S. cerevisiae*, *C. albicans*, *G. lucidum*, *V. volvacea*, *P. ostreatus*, and *C. neoformans*, were used. The native promoter and terminator regions of *cpc-2* from *N. crassa* were used as the promoter region and terminator region, respectively. The plasmid with *cpc-2* of *N. crassa* was used as the positive control.

A schematic representation of the complementing construction is shown in Supplementary Figure S1. A 1551 bp downstream regulatory region of *cpc-2* was amplified by PCR with primer pair cpc2-DS-F/R from the genomic DNA of *N. crassa* strain FGSC#4200 and inserted into pCB1532 by homologous recombination (ClonExpress MultiS One Step Cloning Kit; Vazyme, Nanjing, China) to generate plasmid pre-pCB1532-cpc2. This plasmid was used as a precursor in the following construction.

To construct the complementing vector with *N. crassa cpc-2*, the full length of *cpc-2* (3459 bp) was amplified by PCR with primer pair cpc2-CM-F/R from the genomic DNA of *N. crassa* strain FGSC#4200. The PCR product was introduced into pre-cpc2-pCB1532 by homologous recombination to generate the final vector Nccpc-2-pCB1532.

To construct the complementing vector with *Fvcpc2* of *F. velutipes*, the *cpc-2* promoter and terminator were amplified by PCR using primer pairs NCcpc2-Promoter-F/R and NCcpc2-Terminator-F/R from the genomic DNA of *N. crassa* strain FGSC#4200, respectively. The open reading frame region of *Fvcpc2* (948 bp) was amplified by PCR with primer pair Fvcpc2-F/R from the cDNA of *F. velutipes* strain L11. These three fragments were inserted into pre-cpc2-pCB1532 by homologous recombination to obtain the final plasmid, Fvcpc2-pCB1532. The construction of plasmids Sccpc2-pCB1532, Caasc1-pCB1532, Glcpc2-pCB1532, Cngib2-pCB1532, Vvcpc2-pCB1532, and Pocpc2-pCB1532 was performed following the same method as for Fvcpc2-pCB1532. These plasmids were confirmed by PCR and sequencing. The primers employed for plasmid construction were shown in Supplementary Table S1.

The above accomplished plasmids were transferred into the Δ*cpc-2* strain via electroporation (Margolin et al., 1997). Then the transformants were selected by chlorimuron ethyl (15 μg ml^–1^) and confirmed with PCR. Finally, the complemented strains with *cpc-2* of *N. crassa* and CPC-2 ortholog encoding genes from *F. velutipes*, *S. cerevisiae*, *C. albicans*, *G. lucidum*, *V. volvacea*, *P. ostreatus*, and *C. neoformans* were achieved and were named as *cpc-2*;Δ*cpc-2*, *Fvcpc2*;Δ*cpc-2*, *cpc2*;Δ*cpc-2*, *asc1*;Δ*cpc-2*, *Glcpc2*;Δ*cpc-2*, *Vvcpc2*;Δ*cpc-2*, *Pocpc2*;Δ*cpc-2*, and *gib2*;Δ*cpc-2*, respectively. The primers for verifying complemented strains are shown in Supplementary Table S1.

### Measurement of Hyphal Growth Rate and Conidial Production

The growth rate of *N. crassa* in race tubes filled with Vogel’s minimal medium was measured as previously described (Davis and Serres, 1970). The fungal growth of 4 days was recorded per 12 h by marking the visible colony edge on race tubes. Then the hyphal growth rate was calculated as average hyphal extension per day. Three replications were taken independently.

The conidial production of *N. crassa* was measured in test tubes each containing 2 ml Vogel’s solid medium. A mycelial plug (*d* = 5 mm) of wild type FGSC#4200 or *cpc-2* mutants was inoculated into one test tube. After incubation at 28°C for 10 days, spores in each test tube were suspended into 2 ml sterile water. Then conidia were counted with hemocytometer under a BX51 microscope (Olympus, America).

### Crosses and Fertility Tests of *N. crassa*

The crosses and fertility tests were performed in *N. crassa* using standard techniques (Davis and Serres, 1970). The female parents were inoculated on the plates first. Specifically, the conidial suspension (2 μl, 1 × 10^7^ spores ml^–1^) was spotted onto the center of SCM plate and plates were grown at 25°C in dark for 7 days. Then 2 μl conidial suspension of wild-type strain (FGSC#2225) with the opposite mating type A was spotted on the colony surface of the female parent strains, respectively. The crossed plates were incubated at 25°C in dark for another 7 days to induce the formation of perithecia.

### Plasmid Construction and Transformation in *F. velutipes*

The binary vector pBHg-BCA1 containing the resistance gene of hygromycin was used in the construction of the *Fvcpc2* overexpression plasmid and RNAi plasmid as previously described (Wu et al., 2019). The promoter of glyceraldehyde-3-phosphate dehydrogenase P*gpd* and terminator of *trpC* (T*trpC*) were used to control the expression of *Fvcpc2* or the sense and antisense sequence of *Fvcpc2* in both plasmids (Kuo et al., 2004; Sekiya et al., 2013; Kim et al., 2015).

To construct the overexpression plasmid, P*gpd* and T*trpC* were first amplified by PCR with the primer pairs Pgpd-F/R and TtrpC-F/R from the genomic DNA of *F. velutipes* strain L11 and plasmid pCSN44 (Fungal Genetics Stock Center^1^; University of Kansas Medical Center), respectively (Nishikawa et al., 2016). The open reading frame region of *Fvcpc2* (1273 bp) was then amplified by PCR with the primer pair pdd1-F/R from the genomic DNA of strain L11. The above three fragments were introduced into pBHg-BCA1. Finally, the overexpression plasmid, Fvcpc2-OE, was achieved.

To construct the *Fvcpc2* RNAi plasmid, P*gpd* and T*trpC* were first amplified as described above. Then, a 340 bp antisense sequence of *Fvcpc2* was obtained from *F. velutipes* L11 by PCR with primer pair Fvcpc2-antisense-F/R. These three fragments were inserted into pBHg-BCA1 to form the vector pre-Fvcpc2-RNAi. After that, the sense sequence linked with the spacer (from the 113rd nucleotide to the 502nd nucleotide, 390 bp) was amplified from the genomic DNA of strain L11 by PCR with primer pair Fvcpc2-sense-F/R, and ligated by *Bam*HI (New England Biolabs, Beverly, MA, United States) into pre-Fvcpc2-RNAi to produce the final Fvcpc2-RNAi vector. The primers for plasmid construction were shown in Supplementary Table S2.

Plasmids Fvcpc2-OE and Fvcpc2-RNAi were transformed into *F. velutipes* dikaryotic strain F19 by the *A. tumefaciens*-mediated transformation method (Wu et al., 2019), respectively. The positive transformants were detected by PCR with primers Pgpd-detect-F and TtrpC-detect-R. The primers for transformant verification are shown in Supplementary Table S2.

### Fruiting Body Production in *F. velutipes*

The fruiting body was produced on the composted sawdust substrate (Wu et al., 2019). In order to mimic the industrial production of Winter Mushroom, the parts of fruiting bodies out of the vessel were harvested and the fresh weight was used to record the yield of each vessel. Biological efficiency for each strain was calculated by the formula: BE (%) = (weight of fresh basidiomata/weight of dry substrate) × 100 (Harith et al., 2014; Xie et al., 2017). All the results were obtained by measuring basidioma harvested from six vessels for each strain and the average values were then calculated. These cultivation experiments were implemented for three times independently.

### RNA Extraction and Gene Transcription Analysis

To obtain *N. crassa* samples at vegetative growth stage for RNA extraction, conidial suspension (2 μl, 1 × 10^7^ spores ml^–1^) of each strains (FGSC#4200, Δ*cpc-2*, *cpc-2*;Δ*cpc-2*, and *Fvcpc2*;Δ*cpc-2*) was spotted on the center of the cellophane-covered Vogel’s plates and incubated at 28°C for 24 h. After 24 h, mycelia were collected for RNA extraction. To obtain *N. crassa* samples at sexual developmental stage, conidia were spotted on the center of the cellophane-covered SCM plates and incubated at 25°C for 5 days in dark. After 5 days, the mycelia were collected for RNA extraction. The mycelial samples were frozen in liquid nitrogen and ground into fine powder.

To detect the *Fvcpc2* transcript levels in *F. velutipes*, the samples were prepared following the method below. Mycelial plugs (*d* = 5 mm) of strains (F19, *Fvcpc2* overexpression strains and *Fvcpc2* RNAi strains) were respectively inoculated on the center of cellophane-covered CYM agar plates at 25°C. After 7 days, mycelia were harvested for RNA extraction.

To detect the genes responsive to *Fvcpc2* knockdown in *F. velutipes*, the mycelia of wild type strain F19 and *Fvcpc2* knockdown mutants were harvested from composted sawdust substrate (SM) after cold stimulation for fruiting body development.

The harvested mycelia were frozen in liquid nitrogen and ground into fine powder. Total RNA extraction was performed by the standard TRIzol protocol (Invitrogen, Carlsbad, CA, United States). Then 2 μg of total RNA were used to synthesize cDNAs by a cDNA synthesis kit (FastQuant RT Kit with gDNase, TIANGEN, China) according to the manufacturer’s protocol. Quantitative real-time PCR (qPCR) was implemented on a CFX96 multicolor real-time PCR detection system (Bio-Rad, Hercules, CA, United States) with SYBR green detection (KAPA SYBR^®^ FAST qPCR Kits; KAPA BIOSYSTEMS, Boston, MA, United States) following the manufacturer’s instructions. Three independent replicates were performed for each cDNA sample and the average threshold cycle was calculated. The expression of genes was normalized to that of β*-tublin* in *N. crassa*. The expression of genes was normalized to that of β*-actin* in *F. velutipes* (Tao et al., 2016). The 2^–ΔΔCt^ calculation was used for the calculation of relative expression levels (Livak and Schmittgen, 2001). The significance of the differences between each of two samples were estimated by an unpaired two-tailed Student’s *t*-test using GraphPad Prism 7.0 and a *p*-value of less than 0.05 was considered as significant. The primers employed for qPCR are shown in Supplementary Table S3.

## Results

### Identification of the CPC-2 Ortholog FvCPC2 in *F. velutipes*

**FIGURE 1:**
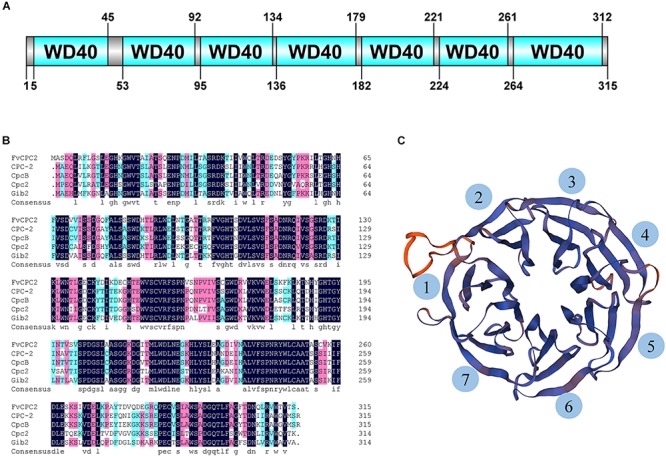
Sequence and structure analysis of FvCPC2 protein. **(A)** The domain organization of FvCPC2 protein. Seven WD40 repeats were predicted in FvCPC2 protein by SMART (http://smart.embl-heidelberg.de/). **(B)** The Sequence alignment of FvCPC2, CPC-2, CpcB, Cpc2, and Gib2 in WD repeats domain. **(C)** The structural model of FvCPC2. The structural model of FvCPC2 was compared with Gib2 from *Cryptococcus neoformans* by SWISS-MODEL (https://www.swissmodel.expasy.org/).

CPC-2 of *N. crassa* belongs to the WD40 protein family. A total of 51 WD40 proteins were predicted in the genome of *F. velutipes* strain L11, however, only one CPC-2 ortholog was predicted. A gene coding the CPC-2 ortholog was found in the genome of *F. velutipes* and named as *Fvcpc2* (GenBank Accession No. KY815023). The genome sequence of *Fvcpc2* is 1273 bp containing an open reading frame of 948 bp interrupted by four introns of 50, 50, 177, and 48 bp, respectively. *Fvcpc2* encodes a protein with 315 amino acid residues which displays 73% amino acid identity with CPC-2 of *N. crassa* (99% coverage). In addition, the amino acid sequence of FvCPC2 also shares highly identity with CpcB of *A. nidulans*, Cpc2 of *S. pombe* and Gib2 of *C. neoformans* (Figure 1B). Domain prediction with SMART showed that FvCPC2 was exclusively built up of seven WD40 repeats which formed seven β-propeller blades in three-dimensional structure (Figures 1A,C).

**FIGURE 2:**
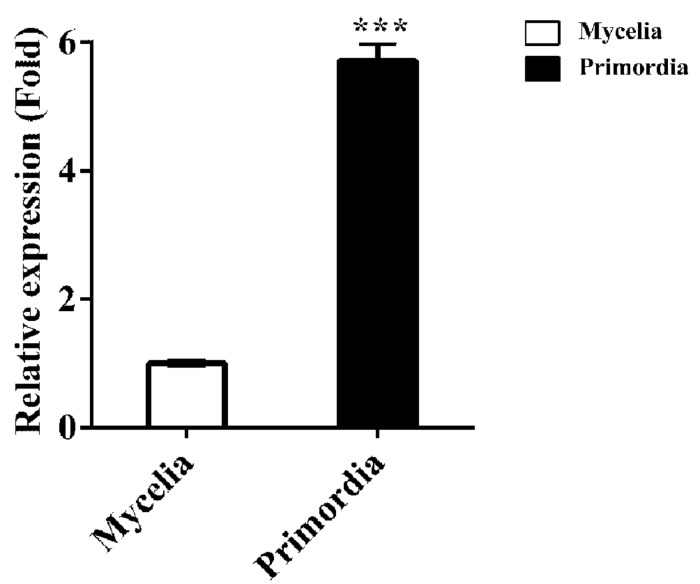
Transcript analysis of *Fvcpc2* in *F. velutipes*. RNA was extracted from the vegetative mycelia growing in composted sawdust substrate (Mycelia) and primordia (Primordia) in wild-type strain F19. The transcript levels of *Fvcpc2* were measured by qPCR. The results presented in the figure are means of three biological replicates with error bars and the significant level between primordia and vegetative mycelia was calculated by *t*-test and marked by *** (*p* < 0.001). (*p* = 0.0000026, *n* = 3).

qPCR analysis showed that the transcriptional level of *Fvcpc2* in primordia were 5.71-fold more than in mycelia at the vegetative growth stage (Figure 2; *p* = 0.0000026, *n* = 3), consistent with the previously reported RNA-seq data (Liu et al., 2015), suggesting that FvCPC2 is likely to participate in fruiting body development.

### CPC-2 Orthologs From Seven Fungal Species Can Functionally Replace the Role of *N. crassa* CPC-2 in Vegetative Growth and Asexual Sporulation

**FIGURE 3:**
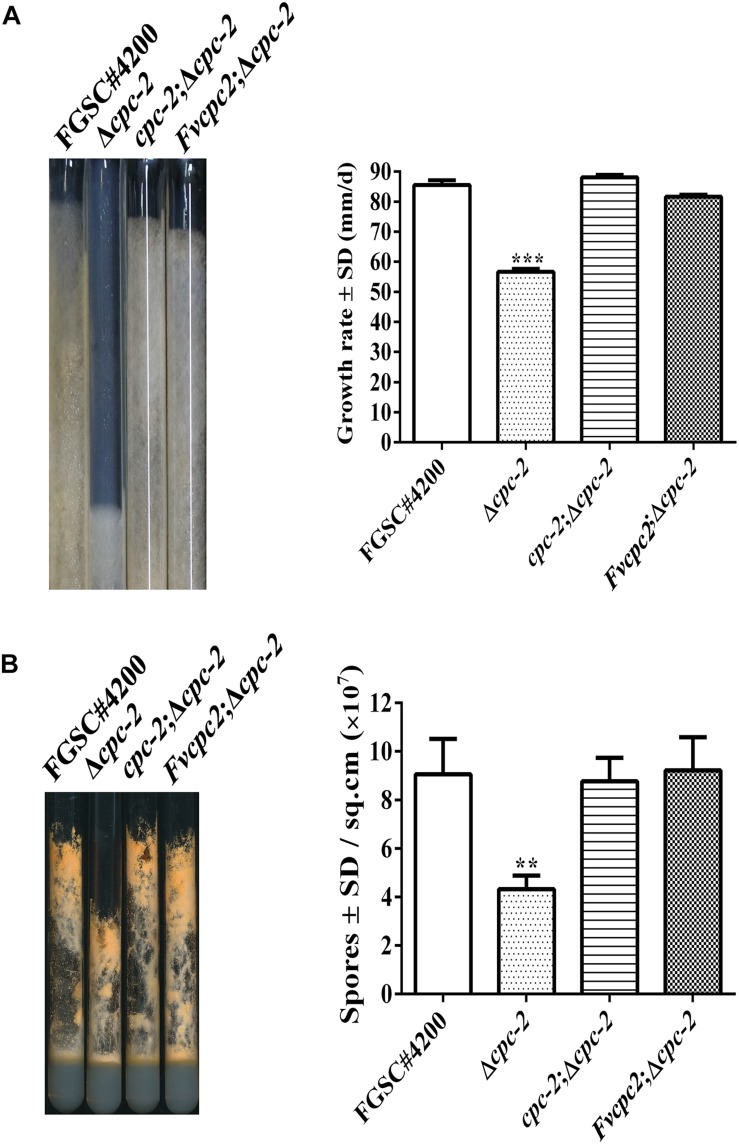
Complementation of *N. crassa* Δ*cpc-2* with *Fvcpc2* in growth and conidial production. **(A)** Strains were grown in race tubes and their growth rates were calculated based on 4 days of growth at 28°C. **(B)** Strains were inoculated into test tubes containing Vogel’s agar medium and cultured at 28°C. After 10 days, images of test tubes were captured and conidia produced in each tube were calculated. The strains include wild-type strain FGSC#4200, *cpc-2* deletion mutant (Δ*cpc-2*) and complemented mutants (*cpc-2*;Δ*cpc-2* and *Fvcpc2*;Δ*cpc-2*). Values shown are means of three replicates. Standard deviations are indicated with error bars. The significant levels of Δ*cpc-2* in growth rate and conidiation were calculated to wild-type FGSC#4200 by *t*-test and marked as ** (0.001 < *p* < 0.01) or *** (*p* < 0.001).

In order to test whether FvCPC2 are functionally similar to *N. crassa* CPC-2, a strain (*Fvcpc2*;Δ*cpc-2*) was created, in which *Fvcpc2* of *F. velutipes* was transformed into the *cpc-2* deletion mutant (Δ*cpc-2*) of *N. crassa* (Supplementary Figure S1). Meanwhile, another strain (*cpc-2*;Δ*cpc-2*), in which *N. crassa cpc-2* was transformed into the Δ*cpc-2* mutant and used as a control strain. Similar to the previous report (Garud et al., 2019), the Δ*cpc-2* strain grew slower and produced less conidia than *N. crassa* wild type. Both the *Fvcpc2*;Δ*cpc-2* strain and the *cpc-2*;Δ*cpc-2* strain displayed the wild-type colony growth rate and wild-type conidial production ability (Figure 3), indicating *Fvcpc2* can functionally replace the role of *N. crassa* CPC-2 in vegetative growth and asexual sporulation.

We further respectively transformed the genes encoding *cpc-2* orthologs from 6 other fungal species, including *S. cerevisae*, *C. albicans*, *C. neoformans*, *V. volvacea*, *P. ostreatus*, and *G. lucidum*, into the Δ*cpc-2* mutant (Supplementary Figure S2). The sequence conservation of these CPC-2 orthologs was shown in Supplementary Figure S3. All transformants displayed the wild-type phenotype in colony growth and conidial production (Supplementary Figure S2), demonstrating that the CPC-2 orthologs from a wide range of fungal species across Ascomycota and Basidiomycota can functionally replace the role of *N. crassa* CPC-2 in vegetative growth and asexual sporulation.

### CPC-2 Orthologs From Seven Fungal Species Can Functionally Replace the Role of *N. crassa* CPC-2 in Fruiting Body Development

**FIGURE 4:**
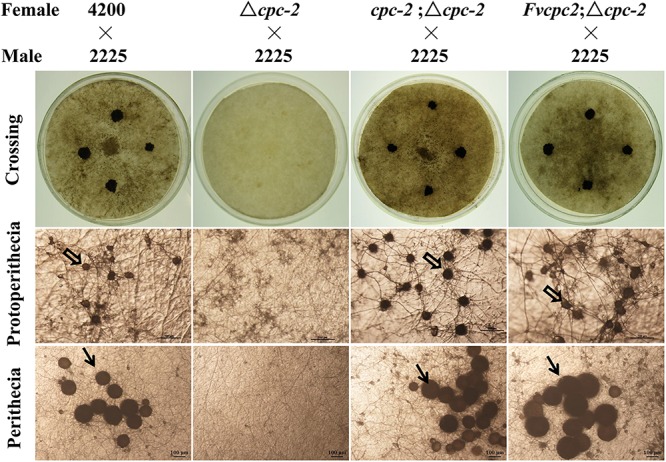
Complementation of *N. crassa* Δ*cpc-2* with *Fvcpc2* in fruiting body development. The wild-type strain FGSC#4200, the *cpc-2* deletion mutant (Δ*cpc-2*) and complemented mutants (*cpc-2*;Δ*cpc-2* and *Fvcpc2*;Δ*cpc-2*) were inoculated on SCM plates and incubated at 25°C in the dark. After 7 days, the opposite mating type strain of wild type (FGSC#2225, mating type A) was spotted on the colony surface of each female strain. Then the plates were cultivated at 25°C under light for another 7 days. The images of protoperithecia and perithecia were captured on the 7th and 14th day by stereomicroscope, respectively. Perithecia and protoperithecia were marked by solid and hollow arrows, respectively. The scale bar is 100 μm.

Fruiting body development in the Δ*cpc-2* mutant of *N. crassa* was blocked and it failed to produce protoperithecium (Müller et al., 1995). We observed the sexual development of all above mentioned strains. As shown in Figure 4, the wild-type strain produced normal protoperithecia after fruiting induction for 3–5 days, while the Δ*cpc-2* strain could form hyphal aggregation but failed to generate protoperithecia. After crossing with a wild-type strain with the opposite mating type, wild type produced abundant perithecia on the colony surface but no perithecium appeared in the Δ*cpc-2* strain (Figure 4). The *cpc-2*;Δ*cpc-2* strain displayed wild-type phenotype and produced normal protoperithecia and perithecia. The *Fvcpc2*;Δ*cpc-2* strain could produce normal protoperithecia and perithecia but the formation of protoperithecia was 2 days later than wild type and the *cpc-2*;Δ*cpc-2* strain. The wild-type strain and the *cpc-2*;Δ*cpc-2* strain formed protoperithecia after three and half days of sexual development induction on the SCM media, while the *Fvcpc2*;Δ*cpc-2* strain needed five and half days.

Similar to the *Fvcpc2*;Δ*cpc-2* strain, all other complement transformants, each of which had one of CPC-2 ortholog genes from six other fungal species in the Δ*cpc-2* background, were able to form protoperithecia and perithecia (Supplementary Figure S4), but the formation of protoperithecia in these strains was delayed 1–4 days compared to wild type and the *cpc-2*;Δ*cpc-2* strain. Nevertheless, above results together indicate that the CPC-2 orthologs can functionally replace the role of *N. crassa* CPC-2 in fruiting body development.

### FvCPC2 Can Functionally Replace the Role of *N. crassa* CPC-2 in Transcriptional Regulation

**FIGURE 5:**
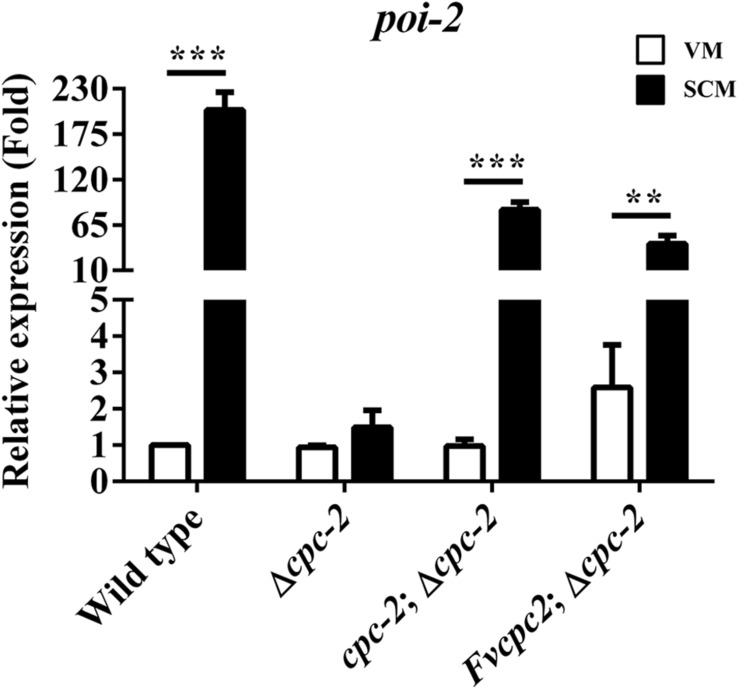
Complementation of *N. crassa* Δ*cpc-2* with *Fvcpc2* in transcriptional regulation of *poi-2*. Transcript levels of *poi-2* were measured at vegetative growth stage (VM) and after fruiting induction (SCM) in wild type (FGSC#4200), *cpc-2* deletion mutant (Δ*cpc-2*) and *cpc-2* complemented mutants (*cpc-2*;Δ*cpc-2* and *Fvcpc2*;Δ*cpc-2*) by qPCR in *N. crassa*. The results presented are means of three biological replicates with error bars and significant level was calculated by *t*-test and marked as ** (0.001 < *p* < 0.01) or *** (*p* < 0.001). P*_poi–__2_*, _wild type_ = 7.6355E-05, *n* = 3; P*_poi–__2_*, *_cpc–__2_*_;__Δ_ *_cpc–__2_* = 9.0510E-05, *n* = 3; P*_poi–__2_*, *_Fvcpc__2_*_;__Δ__c_*_pc–__2_* = 0.0023, *n* = 3.

We detected the expression of more than 70 genes which were reported in fruiting body development in *N. crassa* by qRT-PCR (Kim and Nelson, 2005; Sun et al., 2012, 2019; Garud, 2013; Chinnici et al., 2014; Lehr et al., 2014; Wang et al., 2014), and found that *poi-2*, an essential gene for female fertility in *N. crassa*, had a dramatic response to *cpc-2* deletion (more than 100-fold change) after fruiting induction in *N. crassa* (Figure 5), indicating that CPC-2 regulates transcription of *poi-2*. To see whether FvCPC2 can functionally replace the role of CPC-2 in transcriptional regulation, transcript levels of *poi-2* were analyzed by qPCR in wild type, the Δ*cpc-2* strain, the *cpc-2*;Δ*cpc-2* strain and the *Fvcpc2*;Δ*cpc-2* strain. As shown in Figure 5, *poi-2* had a significant transcriptional increase in response to fruiting induction. However, its significant transcriptional increase did not appear in the Δ*cpc-2* strain. In contrast, both the *cpc-2*;Δ*cpc-2* strain and the *Fvcpc2*;Δ*cpc-2* strain had the significant transcriptional response in *poi-2* to fruiting induction (Figure 5), indicating *F. velutipes Fvcpc2* can functionally replace the role of *cpc-2* in transcriptional regulation of *poi-2* in *N. crassa*.

### Generation of Mutants for *Fvcpc2* Overexpression and Knockdown in *F. velutipes*

Above results suggests that CPC-2 orthologs among the fungal species from the Ascomycota phylum and the Basidiomycota phylum are functionally conserved in fruiting body development. To understand the function of *Fvcpc2* in *F. velutipes*, the *Fvcpc2* overexpression vector Fvcpc2-OE and the *Fvcpc2* knockdown vector Fvcpc2-RNAi were constructed, respectively. The binary vector pBHg-BCA1, the promoter of glyceraldehyde-3-phosphate dehydrogenase (P*gpd*) (Kuo et al., 2004) and the terminator of *trpC* (T*trpC*) (Sekiya et al., 2013; Kim et al., 2015) were used to construct these plasmids as previously described (Wu et al., 2019).

**FIGURE 6:**
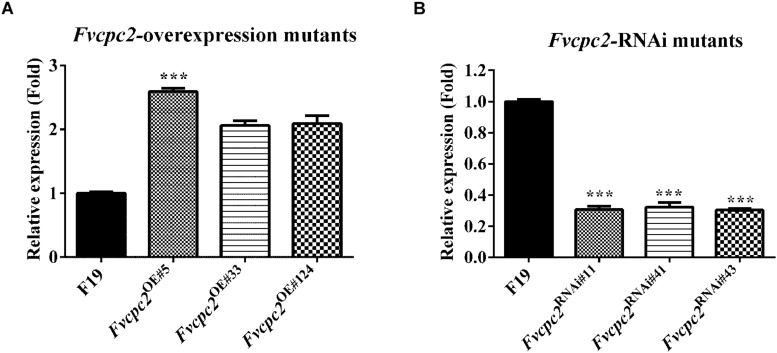
Effects of *Fvcpc2* overexpression and knockdown in *Fvcpc2* transcription. **(A,B)** Transcript levels of *Fvcpc2* in wild type, *Fvcpc2* overexpression strains and *Fvcpc2* knockdown strains. RNA was extracted from the mycelia cultured on cellophane-covered CYM agar at 25°C for 7 days. Transcript levels of *Fvcpc2* were measured by qPCR. The results presented in the figure are means of three biological replicates with error bars. The significant levels of *Fvcpc2* mutants compared to wild type were calculated by *t*-test and marked as *** (*p* < 0.001). (p*_Fvcpc__2_*_OE#__5_ = 0.0005, *n* = 3; p*_Fvcpc__2_*_OE#__33_ = 0.0023, *n* = 3; p*_Fvcpc__2_*_OE#__124_ = 0.0062, *n* = 3; p*_Fvcpc__2_*_RNAi#__11_ = 0.0005, *n* = 3; p*_Fvcpc__2_*_RNAi#__41_ = 0.0009, *n* = 3; p*_Fvcpc__2_*_RNAi#__43_ = 2.4172E-05, *n* = 3).

The two vectors were introduced into a dikaryotic wild-type *F. velutipes* strain F19, respectively. Three *Fvcpc2* overexpression strains *Fvcpc2*^OE#5^, *Fvcpc2*^OE#33^, and *Fvcpc2*^OE#124^ were obtained, in which *Fvcpc2* transcript levels were increased to 2.59-, 2.06-, and 2.09-fold, respectively, relative to the wild-type strain F19 (p*_*Fvcpc*__2_*_*OE#*__5_ = 0.0005, *n* = 3; p*_*Fvcpc*__2_*_*OE#*__33_ = 0.0023, *n* = 3; p*_*Fvcpc*__2_*_*OE#*__124_ = 0.0063, *n* = 3) (Figure 6A). Three *Fvcpc2* knockdown mutants *Fvcpc2*^RNAi#11^, *Fvcpc2*^RNAi#41^, and *Fvcpc2*^RNAi#43^, were obtained, in which *Fvcpc2* transcript levels were decreased by 69.4, 67.7, and 69.7%, respectively, relative to F19 (p*_*Fvcpc*__2_*_*RNAi#*__11_ = 0.0005, *n* = 3; p*_*Fvcpc*__2_*_*RNAi#*__41_ = 0.0009, *n* = 3; p*_*Fvcpc*__2_*_*RNAi#*__43_ = 2.4172E-05, *n* = 3) (Figure 6B). The six strains were used in the following study.

### FvCPC2 Positively Regulates Vegetative Growth in *F. velutipes*

**FIGURE 7:**
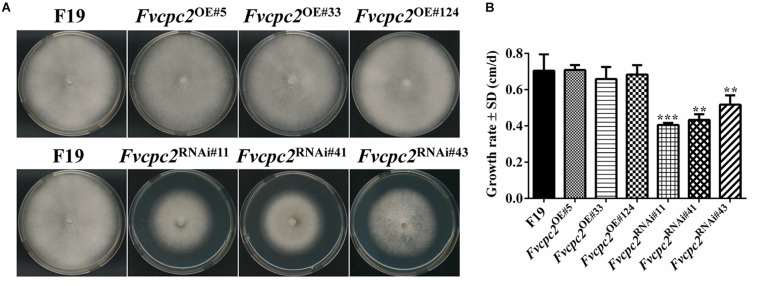
Effects of *Fvcpc2* overexpression and knockdown in vegetative growth on agar plates. **(A)** Mycelial plugs (*d* = 5 mm) of wild type (F19), *Fvcpc2* overexpression strains (*Fvcpc2*^*OE#*5^, *Fvcpc2*^*OE#*33^, and *Fvcpc2*^*OE#*124^) and *Fvcpc2* knockdown strains (*Fvcpc2*^RNAi#11^, *Fvcpc2*^RNAi#41^, and *Fvcpc2*^RNAi#43^) were inoculated onto the center of CYM plates and cultured at 25°C for 7 days, respectively. **(B)** Growth rates of wild type (F19), *Fvcpc2* overexpression and knockdown mutants. The colony edge was marked each 24 h and the growth rates were calculated based on 7 days of growth. Values shown are means of three biological replicates. Standard deviations are indicated with error bars. The significant levels of *Fvcpc2* mutants in growth rate were calculated to wild type by *t*-test and marked as ** (0.001 < *p* < 0.01) or *** (*p* < 0.001). (p*_Fvcpc__2_*_OE#__5_ = 0.6892, *n* = 3; p*_Fvcpc__2_*_OE#__33_ = 0.4876, *n* = 3; p*_Fvcpc__2_*_OE#__124_ = 0.8501, *n* = 3; p*_Fvcpc__2_*_RNAi#__11_ = 7.2068E-05, *n* = 3; p*_Fvcpc__2_*_RNAi#__41_ = 0.0025, *n* = 3; p*_Fvcpc__2_*_RNAi#__43_ = 0.0022, *n* = 3).

When grown on CYM, all three *Fvcpc2* overexpression strains did not display obvious difference from the wild-type strain F19 in colony growth, mycelial density and colony color (Figure 7A). However, the colony growth of *Fvcpc2* knockdown strains, especially *Fvcpc2*^RNAi#41^ and *Fvcpc2*^RNAi#43^, was slower than wild type. As shown in Figure 7B, the growth rates in *Fvcpc2*^RNAi#11^, *Fvcpc2*^RNAi#41^, and *Fvcpc2*^RNAi#43^ were reduced by 42.1 ± 3.3%, 31.6 ± 2.1%, and 21.8 ± 5.8%, respectively, compared to F19 (p*_*Fvcpc*__2_*_*RNAi#*__11_ = 7.2068E-05, *n* = 3; p*_*Fvcpc*__2_*_*RNAi#*__41_ = 0.0025, *n* = 3; p*_*Fvcpc*__2_*_*RNAi#*__43_ = 0.0022, *n* = 3).

**FIGURE 8:**
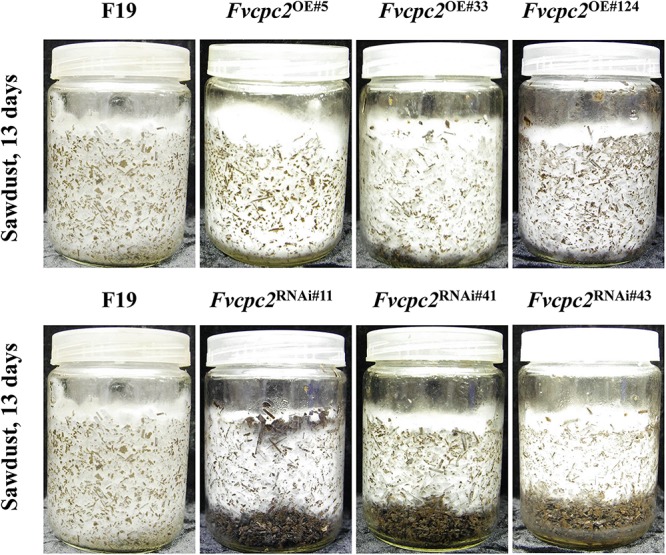
Effects of *Fvcpc2* overexpression and knockdown in vegetative growth in composted sawdust substrate. Wild-type strain F19, *Fvcpc2* overexpression strains (*Fvcpc2*^*OE#*5^, *Fvcpc2*^*OE#*33^, and *Fvcpc2*^*OE#*124^) and *Fvcpc2* knockdown strains (*Fvcpc2*^RNAi#11^, *Fvcpc2*^RNAi#41^, and *Fvcpc2*^RNAi#43^) were inoculated into the culture vessels each containing 150 ± 5 g composted sawdust substrate. The photographs of each strain were captured after 13 days of growth at 25°C.

The difference in vegetative growth between *Fvcpc2* mutants and the wild-type strain F19 was also observed in the composted sawdust substrate. All strains were cultured at 25°C. After 13 days, the mycelia of the wild-type strain and *Fvcpc2* overexpression strains completely occupied the substrate, while the mycelia of three *Fvcpc2* knockdown mutants reached only two-thirds of the vessels (Figure 8). In fact, the mycelia of *Fvcpc2* knockdown mutants needed 8 more days to cover the entire vessel than the wild-type strain. Above results indicate that FvCPC2 plays a positive role in hyphal growth of *F. velutipes*.

### FvCPC2 Positively Regulates Fruiting Body Development in *F. velutipes*

**FIGURE 9:**
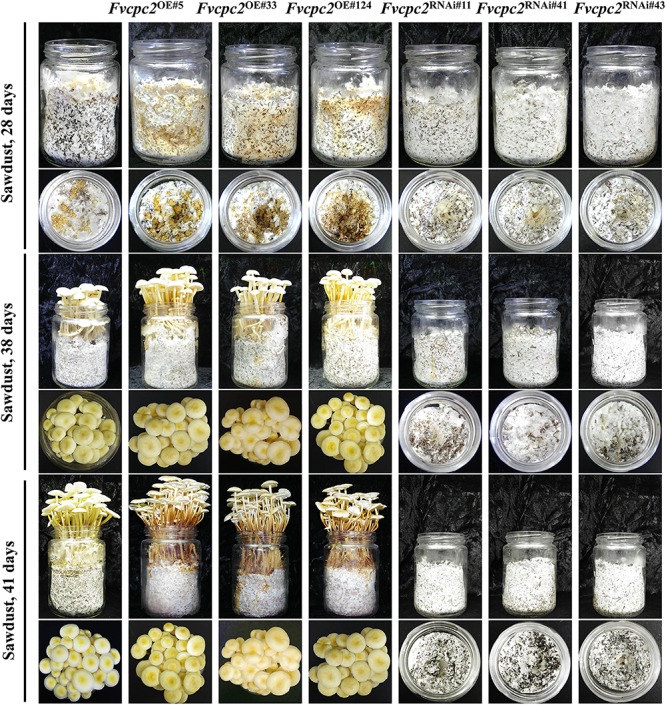
Effects of *Fvcpc2* overexpression and knockdown in fruiting body development. Cultures from a half colony growing on CYM plate (*d* = 60 mm) were punched into mycelial plugs (*d* = 5 mm) and inoculated into the culture vessels each containing 150 ± 5 g composted sawdust substrate. Images of fruiting body development were captured on the 28th, 38th, and 41st days after inoculation. Strains includes wild type (F19), *Fvcpc2* overexpression strains (*Fvcpc2*^*OE#*5^, *Fvcpc2*^*OE#*33^, and *Fvcpc2*^*OE#*124^), and *Fvcpc2* knockdown strains (*Fvcpc2*^RNAi#11^, *Fvcpc2*^RNAi#41^, and *Fvcpc2*^RNAi#43^).

Fruiting body development of *F. velutipes* strains grown in the composted sawdust substrate was observed. After 21 days of cultivation, the mycelial mat on the sawdust surface was scrapped off to stimulate fruiting body development. The primordia in wild type appeared 7 days after stimulation (on the 28th day), while the *Fvcpc2* overexpression strains formed primordia one day earlier than wild type and they obviously produced more primordia than wild type. The optimal time for mushroom harvest in wild type appeared on the 41st day, while the mushrooms in *Fvcpc2* overexpression strains could be harvested 3 days earlier than wild type (Figure 9). Thus, overexpression of *Fvcpc2* could shorten the cultivation time by 7.3%.

All the *Fvcpc2* knockdown strains failed to produce fruiting bodies (Figure 9). Even when the cultivation time was prolonged to 45 days, fruiting body was still not formed. The deficiency of mushroom formation in *Fvcpc2* knockdown strains and the shortened cultivation time in *Fvcpc2* overexpression strains were observed in another two independently repeated experiments (Supplementary Figure S4).

### Overexpression of *Fvcpc2* Increases Mushroom Yield in *F. velutipes*

The fruiting bodies of the wild-type strain and *Fvcpc2* overexpression strains were harvested on the 41st day. As shown in Table 1, *Fvcpc2* overexpression strains did not display obvious difference with the wild-type strain in fruiting body number. The average number of fruiting bodies for wild type and all *Fvcpc2* overexpression strains was about 68 per vessel.

**TABLE 1:** The mushroom yield in wild type (F19) and *Fvcpc2* overexpression strains.

**Strains**	**Average fruiting body number*^a^***			**Average stipe height (cm)**			**Average yield (g per vessel)**			**Average biological efficiency (%)**		
	**R1^b^**	**R2**	**R3**	**R1**	**R2**	**R3**	**R1**	**R2**	**R3**	**R1**	**R2**	**R3**
F19	69 ± 2	68 ± 2	67 ± 2	6.95 ± 0.12	6.70 ± 0.09	6.79 ± 0.16	23.37 ± 0.85	23.42 ± 0.75	23.05 ± 0.60	45.35 ± 1.67	45.03 ± 1.45	44.33 ± 1.15
*Fvcpc2*^*OE#5*^	69 ± 2	67 ± 2	67 ± 3	7.98 ± 0.07*^e^*	7.60 ± 0.28*^e^*	7.79 ± 0.23*^e^*	26.57 ± 0.69*^e^*	26.49 ± 1.88*^d^*	26.57 ± 0.70*^e^*	51.09 ± 1.32*^e^*	50.93 ± 3.62*^d^*	51.09 ± 1.34*^e^*
*Fvcpc2*^*OE#33*^	65 ± 2	68 ± 2	68 ± 3	7.76 ± 0.13*^e^*	7.51 ± 0.11*^e^*	7.64 ± 0.19*^e^*	24.90 ± 0.70*^e^*	25.11 ± 0.81*^d^*	25.74 ± 1.49*^d^*	47.89 ± 1.36*^e^*	48.29 ± 1.55*^d^*	49.50 ± 2.87*^d^*
*Fvcpc2*^*OE#124*^	68 ± 1	68 ± 3	67 ± 2	7.88 ± 0.13*^e^*	7.65 ± 0.12*^e^*	7.73 ± 0.21*^e^*	25.49 ± 0.87*^e^*	25.47 ± 1.05*^d^*	26.12 ± 1.17*^e^*	44.94 ± 1.63*^e^*	48.99 ± 2.02*^d^*	50.22 ± 2.24*^e^*

**^*a*^The average fruiting body number, stipe height, yield and biological efficiency were measured in wild type (F19) and Fvcpc2 overexpression strains. ^*b*^The fruiting body experiments were taken three biological repeats (R1, R2, and R3), and every repeat contains six bottles. ^*c*^Significantly different from the wild type, 0.01 < P < 0.05. ^*d*^Significantly different from the wild type, 0.001 < P < 0.01. ^*e*^Significantly different from the wild type, P < 0.001.**

However, the stipe height of *Fvcpc2* overexpression strains (*Fvcpc2*^OE#5^, *Fvcpc2*^OE#33^, and *Fvcpc2*^OE#124^) was significantly increased compared to wild type. The average height of stipes in the wild-type strain was 6.95 ± 0.12 cm, while the average height of stipes in the *Fvcpc2*^OE#5^, *Fvcpc2*^OE#33^, and *Fvcpc2*^OE#124^ strains was 7.98 ± 0.07 cm, 7.76 ± 0.13 cm, 7.88 ± 0.13 cm, respectively (Table 1). Thus, the height of stipes could be increased by 14.8% in the *Fvcpc2*^OE#5^ strain which has the highest expression of *Fvcpc2* among these *Fvcpc2* overexpression strains.

The yield and biological efficiency were significantly also increased in *Fvcpc2* overexpression strains relative to wild type. As shown in Table 1, the average yield per vessel in the wild-type strain, *Fvcpc2*^OE#5^, *Fvcpc2*^OE#33^, and *Fvcpc2*^OE#124^ was 23.37 ± 0.85 g, 26.57 ± 0.69 g, 24.90 ± 0.70 g, and 25.49 ± 0.87 g, respectively. The strain (*Fvcpc2*^OE#5^) with the highest expression level of *Fvcpc2* had the highest yield and biological efficiency, both of which were increased by 13.69% relative to wild type. Among three independently repeated experiments, the yield was increased at least by 6.57% in the *Fvcpc2*^OE#5^ strain (Table 1).

### *Fvcpc2* Regulates Genes Involved in cAMP Signaling Pathway in *F. velutipes*

**FIGURE 10:**
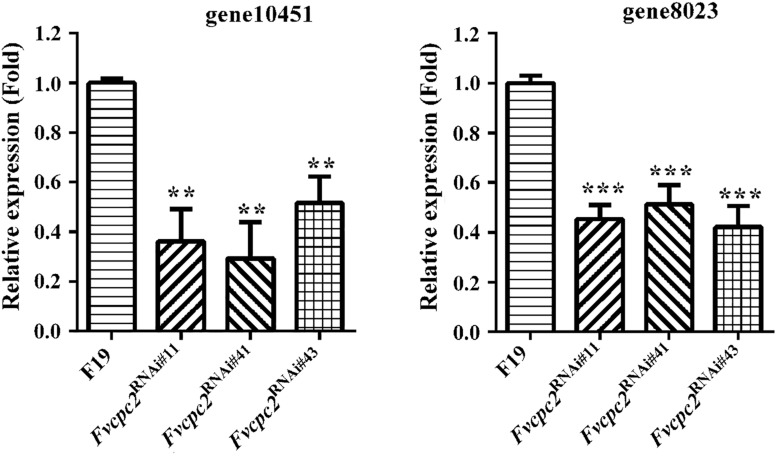
Transcriptional responses of gene10415 and gene8023 to *Fvcpc2* knockdown. Transcript levels of gene10415 (coding adenylate cyclase) and gene8023 (coding kinase A catalytic subunit-2) were determined in wild type (F19) and the *Fvcpc2* knockdown strain *Fvcpc2*^RNAi#43^ by qPCR after fruiting induction in *F. velutipes*. The results presented are means of three biological replicates with error bars and significant level of the indicated genes in *Fvcpc2*^RNAi#43^ compared to wild type were calculated by *t*-test and marked as ** (0.001 < *p* < 0.01) or *** (*p* < 0.001). In *Fvcpc2*^RNAi#11^, p_gene__10451_ = 0.0010, *n* = 3; p_gene__8023_ = 7.7599E-05, *n* = 3. In *Fvcpc2*^RNAi#41^, p_gene__10451_ = 0.0011, *n* = 3; p_gene__8023_ = 0.0004, *n* = 3. In *Fvcpc2*^RNAi#43^, p_gene__10451_ = 0.0014, *n* = 3; p_gene__8023_ = 0.0003, *n* = 3.

The CPC-2 ortholog Gib2 in *C. neoformans* functions as a typical Gβ subunit, which regulates the cellular cAMP (cyclic AMP) level (Palmer et al., 2006). The cAMP level mediates vegetative growth and fruiting body formation in *C. cinerea*, *S. commune* and *V. volvacea* (Kinoshita et al., 2002; Kües et al., 2004; Lu et al., 2015). In order to detect whether *Fvcpc2* is related to cAMP production, transcript levels of gene10451 and gene8023, which respectively encode adenylate cyclase and protein kinase A catalytic subunit-2, were comparatively analyzed in all *Fvcpc2* knockdown strains and the wild-type strain after fruiting induction in *F. velutipes*. In all *Fvcpc2* knockdown strains, transcript levels of both gene10451 and gene8023 were significantly lower than wild type (Figure 10).

**FIGURE 11:**
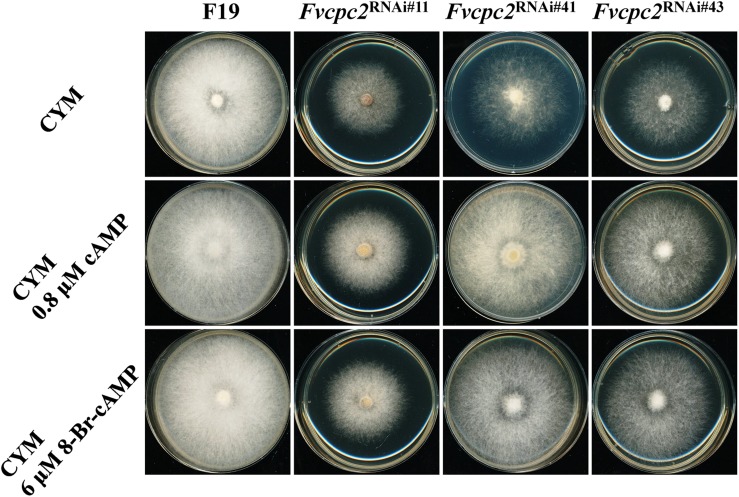
Effects of cAMP or 8-Bromo-cAMP addition in colony growth of *F. velutipes* WT and *Fvcpc2* knockdown strains. Mycelial plugs (*d* = 5 mm) of wild type (F19) and *Fvcpc2* knockdown strains (*Fvcpc2*^RNAi#11^, *Fvcpc2*^RNAi#41^, and *Fvcpc2*^RNAi#43^) were inoculated onto the center of CYM plates with or without 0.8 μM cAMP or 6 μM 8-Bromo-cAMP (8-Br-cAMP), respectively. The plates were cultured at 25°C for 7 days for photography.

The reduced expression of adenylate cyclase and protein kinase A catalytic subunit-2 might result in an insufficient supply of cAMP for normal hyphal growth. To test this possibility, effects of cAMP and the PKA-activator 8-Bromo-cAMP in colony growth of wild type and all *Fvcpc2* knockdown strains were observed (Figure 11). When 0.8 μM cAMP or 6 μM 8-Bromo-cAMP was added into the CYM plate, the colony growth of wild type was not obviously affected. However, the colony of all *Fvcpc2* knockdown strains obviously promoted by addition of cAMP or 8-Bromo-cAMP. Their colonies grew faster on plates amended with cAMP or 8-Bromo-cAMP than on control plates, further suggesting that the slower growth phenotype in these mutants might be due to insufficient production of cAMP.

### FvCPC2 Positively Regulates Four Lectin Genes in *F. velutipes*

To understand how FcCPC2 regulates the fruiting body development, transcriptional levels of some genes related to basidioma development, were comparatively analyzed by qPCR in wild type and the *Fvcpc2* knockdown strain (*Fvcpc2*^RNAi#43^) with the lowest *Fvcpc2* expression after fruiting induction.

**FIGURE 12:**
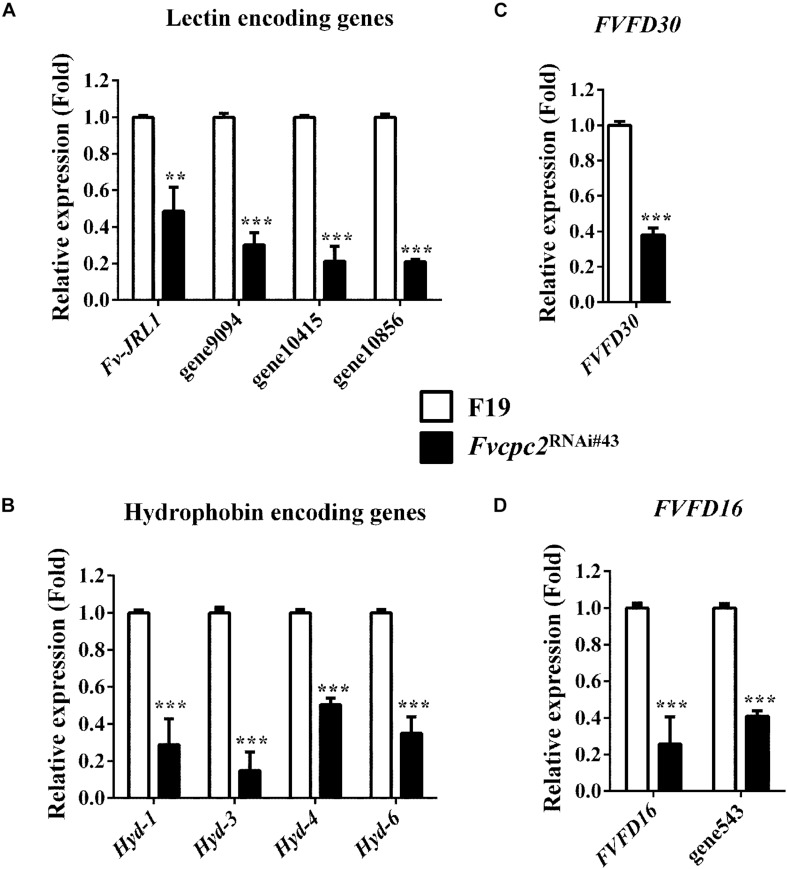
Impacts of *Fvcpc2* knockdown in transcript levels of genes related to fruiting body development. Transcript levels of genes coding **(A)** lectin (*Fv-JRL1*, gene9094, gene10415, and gene10856), **(B)** hydrophobin (*Hyd1*, *Hyd3*, *Hyd4* and *Hyd6*), **(C)** FVFD30 (*FVFD30*) and **(D)** FVFD16 and its homolog (*FVFD16* and gene543) were determined in wild type (F19) and the *Fvcpc2* knockdown strain *Fvcpc2*^RNAi#43^ by qPCR. Results shown are means of three biological replicates. Standard deviations are indicated with error bars. The significant levels of *Fvcpc2*^RNAi#43^ compared to wild type were calculated by *t*-test and marked as ** (0.001 < *p* < 0.01) or *** (*p* < 0.001). p*_v–JRL__1_* = 0.0025, *n* = 3; p_gene__9094_ = 5.1650E-05, *n* = 3; p_gene__10415_ = 7.2365E-05, *n* = 3; p_gene__10856_ = 5.4826E-08, *n* = 3; p*_Hyd–__1_* = 0.0003, *n* = 3; p*_Hyd–__3_* = 0.0001, *n* = 3; p*_Hyd–__4_* = 1.7168E-05, *n* = 3; p*_Hyd–__6_* = 0.0002, *n* = 3; p*_FVFD__30_* = 1.1865E-05, *n* = 3; p*_FVFD__16_* = 0.0009, *n* = 3; p_gene__543_ = 3.8621E-06, *n* = 3.

According to previous studies, lectins promote hyphal aggregation during fruiting body development of mushrooms (Wang et al., 1998; Kües and Liu, 2000). Nine lectin genes were found in *F. velutipes* and one of them, *Fv-JRL1* (encodes jacalin-related lectin), was previously characterized. Overexpression of *Fv-JRL1* promoted vegetative growth and basidioma development, while knockdown of *Fv-JRL1* impaired basidioma development in *F. velutipes* (Lu et al., 2016). Our analysis showed that 4 of 9 lectin genes had difference in transcriptional levels between wild type and *Fvcpc2*^RNAi#43^. The transcriptional level of *Fv-JRL1* in the *Fvcpc2* knockdown strain was significantly lower than that in wild type (Figure 12A). Transcriptional levels of three other lectin encoding genes (gene9094, gene10415 and gene10856) were also lower in *Fvcpc2*^RNAi#43^ than in wild type. Transcriptional levels of these 4 lectin genes in *Fvcpc2*^RNAi#43^ were reduced at least by 51.47% relative to wild type.

### FvCPC2 Positively Regulates Four Hydrophobin Genes in *F. velutipes*

Ten hydrophobin encoding genes (*Hyd-1* to *Hyd-10*) were specifically expressed in the primordium stage in *F. velutipes* (Kim et al., 2016). We analyzed transcripts of all these genes by qPCR and found that 4 of 10 hydrophobin encoding genes, including *Hyd-1* (also reported as *fv-hyd1*) (Yamada et al., 2005), *Hyd-3*, *Hyd-4*, and *Hyd-6*, had transcriptional responses to the *Fvcpc2* knockdown. As shown in Figure 12B, transcriptional levels of *Hyd-1*, *Hyd-3*, *Hyd-4*, and *Hyd-6* in *Fvcpc2*^RNAi#43^ were reduced at least by 49.23% relative to wild type.

### FvCPC2 Positively Regulates Other Genes Related to Fruiting Body Development

Two genes *FVFD16* and *FVFD30* are specifically expressed during fruiting body development in *F. velutipes* (Kim and Azuma, 1999; Kim et al., 1999). FVFD16 has a homolog encoded by gene543 in *F. velutipes*. We analyzed their transcript levels and found that the transcript levels of *FVFD30*, *FVFD16*, and gene543 were significantly down-regulated in the *Fvcpc2* knockdown strain compared to wild type (Figures 12C,D).

## Discussion

Although fruiting structures are distinct between Ascomycota and Basidiomycota, they are two closely related phyla separated only for about 500 million years ago (Berbee and Taylor, 2010; Oberwinkler, 2012). Two phyla share some common regulatory mechanisms in fruiting body development, including the blue light receptor WC-1 (Linden et al., 1997; Terashima et al., 2005; Chen et al., 2012; Ohm et al., 2013; Yang et al., 2016), the pheromone signaling (Debuchy et al., 2010; Raudaskoski and Kothe, 2010) and the heterotrimeric G-protein complex (Lengeler et al., 2000; Brown and Casselton, 2001; Li et al., 2007). Our study revealed that CPC-2 proteins also mediate fruiting body development in both phyla. Since overexpression of *Fvcpc2* could increase mushroom yield and shorten the cultivation time in Winter Mushroom, this study discovered a new gene for mushroom breeding.

The biological functions of CPC-2 and its orthologs were investigated individually in several different fungal species (Krüger et al., 1990; Müller et al., 1995; Gerbasi et al., 2004; Palmer et al., 2006; Liu et al., 2010; Wang et al., 2011, 2014; Kong et al., 2013; Cai et al., 2015), but it was unknown whether these proteins have the same cellular function. By transforming CPC-2 ortholog encoding genes, from 7 different fungal species across Ascomycota and Basidiomycota, into the *cpc-2* deletion mutant of *N. crassa*, we demonstrate that all tested CPC-2 orthologs are able to complement the defects of the *cpc-2* deletion mutant in sexual development, indicating these CPC-2 orthologs can functionally replace CPC-2 in *N. crassa*. Thus, at the protein level, CPC-2 and its orthologs might have the same cellular function. As WD40 domains provide the platform for protein-protein interaction, proteins interacting with CPC-2 orthologs should be structurally similar to those CPC-2-associated proteins in *N. crassa*. Since *cpc-2* in *N. crassa*, *cpcB* in *A. nidulans* and *Fvcpc2* in *F. velutipes* are required for fruiting body development (Müller et al., 1995; Kong et al., 2013), CPC-2 and its orthologs are functionally conserved in regulation of sexual development in both Ascomycota and Basidiomycota and they are likely to be key regulatory components in morphological development of sexual reproduction. To fully understand their regulatory mechanisms in morphological development, the proteins interacting with CPC-2 orthologs and the signaling pathway (s) involved with CPC-2 orthologs are necessary to be identified in future.

By analyzing transcriptional levels of genes related to fruiting body development in *F. velutipes*, we demonstrate that *Fvcpc2* is required for transcriptional responses to sexual development induction. Although we did not investigate the genome-wide effects of *Fvcpc2* knockdown in gene transcription, our limited data indicate the importance of *Fvcpc2* in transcriptional regulation during morphological development of sexual reproduction in Winter Mushroom. The regulatory mechanism of *Fvcpc2* might be involved in the following aspects.

First, *Fvcpc2* might be required for the normal production of cellular cAMP. The cAMP level mediates vegetative growth and fruiting body formation in *C. cinerea*, *S. commune*, and *V. volvacea* (Kinoshita et al., 2002; Kües et al., 2004; Lu et al., 2015). The second messenger cAMP, produced by adenylate cyclase, is involved in many developmental processes in fungi (Lengeler et al., 2000). Both *Fvcpc2* and *cpc-2* regulated the transcription of genes encoding adenylate cyclase and protein kinase A catalytic subunit, and Gib2 positively regulates the cellular cAMP (cyclic AMP) level in *C. neoformans* (Palmer et al., 2006), suggesting that the regulation of cAMP production is a conserved function of the CPC-2 proteins. Our data suggest that the production of cAMP is insufficient in these *Fvcpc2* knockdown mutants because the *Fvcpc2* knockdown made colony growth slower than wild type and the addition of cAMP or the PKA-activator 8-Bromo-cAMP into the medium restored the *Fvcpc2* knockdown mutants to the wild-type colony growth phenotype. In addition, as Gib2 stabilizes Gpa1 in *C. neoformans* (Palmer et al., 2006), FvCPC2 might function in the stabilization of Gα protein in *F. velutipes* and thus guarantee the normal concentration of cAMP in cells. Therefore, the regulation of cAMP production is likely to be an important regulatory mechanism of FvCPC2 in morphological development. CPC-2 functions as the downstream of GNA-2 and the upstream of GNB-1 in hyphal growth and perithecial development in *N. crassa*, respectively (Garud et al., 2019). Since FvCPC2 has the similar role as CPC-2 of *N. crassa* in vegetative growth and fruiting body development, FvCPC2 might interact with the G protein signaling as CPC-2 does in *F. velutipes*.

Second, FvCPC2 regulates fruiting body development likely through mediating the expression of hydrophobins. Hydrophobins are fungal specific secretory proteins which could self-assemble into amphipathic layers at hydrophilic-hydrophobic interfaces (Wessels, 1996). The formation of amphipathic layers is beneficial for mycelia to form air channels, which probably promote gas exchange for fruiting bodies (Wösten and Wessels, 1997; Wessels, 2000). The important role of several hydrophobin encoding genes in the fruiting body formation have been demonstrated in mushroom species *Agaricus bisporus*, *P. ostreatus*, and *L. edodes* (De Groot et al., 1996; Ásgeirsdóttir et al., 1998; Ng et al., 2000). In *F. velutipes*, ten hydrophobin genes (*Hyd-1* to *Hyd-10*) had increased expression during fruiting body development (Ando et al., 2001; Yamada et al., 2005; Kim et al., 2016). Relatively high transcriptional levels of these hydrophobin genes during fruiting body formation might be necessary for related morphological development. We found that four of them are positively regulated by FvCPC2 and *Fvcpc2* is critical for their normal expression during fruiting body development. Thus, maintenance of transcriptional levels of these four hydrophobin genes by FvCPC2 might play a role in fruiting body development. Loss of regulation by FvCPC2 in these hydrophobin genes might reduce hydrophobin production and further affect mushroom development.

Third, FvCPC2 regulates fruiting body development likely through promoting the expression of lectin genes. Lectin genes are up-regulated in fruiting body development in *Pleurotus cornucopiae*, *C. cinerea*, and *Agrocybe aegerita* (Kaneko et al., 1993; Oguri et al., 1993; Boulianne et al., 2000; Luan et al., 2010). In *Sclerotium rolfsii*, the interaction of lectin with the cell wall-associated putative endogenous lectin receptor promotes the aggregation of mycelia to form sclerotial bodies (Swamy et al., 2004). In *F. velutipes*, the lectin encoding gene *Fv-JRL1* and its three homolog encoding genes were dramatically upregulated at the primordium formation stage (Liu et al., 2015). *F. velutipes Fv-JRL1* positively regulates hyphal growth and fruiting body development (Lu et al., 2016). Thus, enhanced lectin production might be required for normal mushroom formation. Our results showed that reduction of *Fvcpc2* expression could decrease transcription of these lectin genes. Therefore, FvCPC2 plays a critical role in maintaining normal expression of these lectin genes during mushroom development. Transcriptional upregulation of these lectin genes by FvCPC2 might promote lectin production and be important for fruiting body development.

In addition to these genes with relatively clear roles in morphological development, FvCPC2 also regulate other genes with unknown functions. *FVFD16* and *FVFD30* are two genes specifically expressed during fruiting body development of *F. velutipes* (Kim and Azuma, 1999; Kim and Azuma, 2000). Our results showed that *FVFD16*, *FVFD30* and a gene encoding a FVFD16 homolog were downregulated in response to *Fvcpc2* knockdown. Although the functions of these *Fvcpc2*-regulated genes in morphological development remain to be clarified, the regulation of their expression by *Fvcpc2* might have an impact in fruiting body development.

In *N. crassa*, *poi-2* is an essential gene for sexual development. Disruption of *poi-2* caused vegetative growth deficiency and female sterility (Kim and Nelson, 2005). Our results showed that the expression of *poi-2* was increased by about 200-fold after sexual induction. CPC-2 regulates the expression of *poi-2* during sexual development because the increased expression in *poi-2* upon sexual induction disappeared in the *cpc-2* deletion mutant. *Fvcpc2* can replace the role of *cpc-2* in the transcriptional regulation of *poi-2*, suggesting the signal transduction processes involved with CPC-2 proteins are likely conserved among different fungal species. However, the homologs of POI-2 were not hunted by alignment of amino acid sequence in *F. velutipes* and other mushroom species. Thus, the downstream genes regulated by CPC-2 proteins might vary from one fungal species to another.

The capacity of yield and production cost are the core concerns for mushroom industry. In addition to yield increase, overexpression of *Fvcpc2* could also reduce the cultivation time by 3 days in Winter Mushroom. Since the formation of mushrooms for *F. velutipes* needs low temperature (15°C), the production of Winter Mushrooms has high electricity cost and equipment depreciation. The shorter cultivation cycle will dramatically reduce the energy consumption and promote the efficacy of equipment utilization. Thus, *Fvcpc2* can be a promising reference gene for Winter Mushroom breeding. The orthologs of FvCPC2 were highly conserved and widespread in different edible mushrooms. Most of the FvCPC2 ortholog encoding genes specifically expressed during fruiting body development in mushroom species (Yu et al., 2012; Zhang et al., 2015; Sakamoto et al., 2017). Thus, genes encoding FvCPC2 orthologs in other mushroom species may also have potential application in breeding.

## Conclusion

In conclusion, the study demonstrates that CPC-2 and its orthologs are functionally conserved among different fungal species across Ascomycota and Basidiomycota and discovered a new gene with an essential regulatory role in mushroom development and a potential use in mushroom breeding.

## Data Availability Statement

All datasets generated for this study are included in the article/Supplementary Material.

## Author Contributions

SL contributed conception and design of the study. TW performed experiments. SL and TW wrote and edited the manuscript. TW, ZZ, CH, SW, and LZ performed the statistical analysis. All authors contributed to manuscript revision, read and approved the submitted version.

## Conflict of Interest

LZ is employed by Shandong Jinniu Biotech Company Limited. The remaining authors declare that the research was conducted in the absence of any commercial or financial relationships that could be construed as a potential conflict of interest.

## Footnotes

1 www.fgsc.net/

2 https://www.ncbi.nlm.nih.gov/gene/
