# Supplementary material for: A WD40 Protein Encoding Gene Fvcpc2 Positively Regulates Mushroom Development and Yield in Flammulina velutipes
Source: Front Microbiol. 2020 Mar 26;11:498. doi: 10.3389/fmicb.2020.00498 (PMC7113406; doi:10.3389/fmicb.2020.00498)
Supplement: Supplementary file 1 [file Data_Sheet_1.docx]

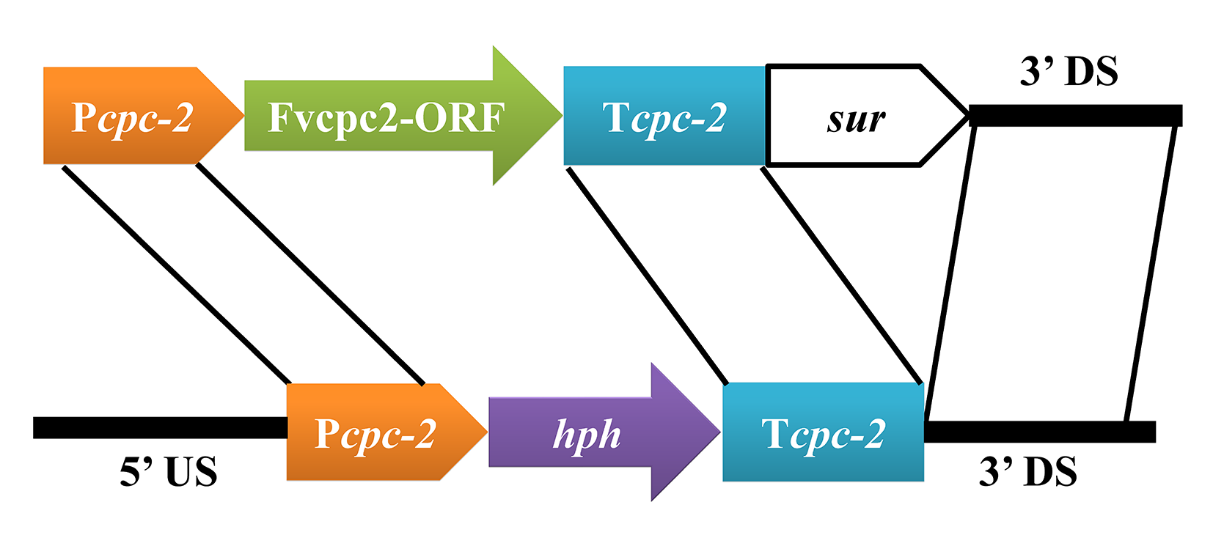
**Figure S1. Schematic diagram of the process of *Fvcpc2*;Δ*cpc-2* strain construction.** Promoter and terminator of *cpc-2* used in the construction are shown as orange arrow and blue rectangle, respectively. The white arrow represents chlorimuron ethyl resistant gene *sur*. The green and purple arrows represent the opening-reading-frame of *Fvcpc2* and *hph* (the hygromycin B hygromycin resistance gene), respectively. 3’ DS and 5’ US represent the 3’ downstream sequence and 5’ upstream sequence of *cpc-2*.


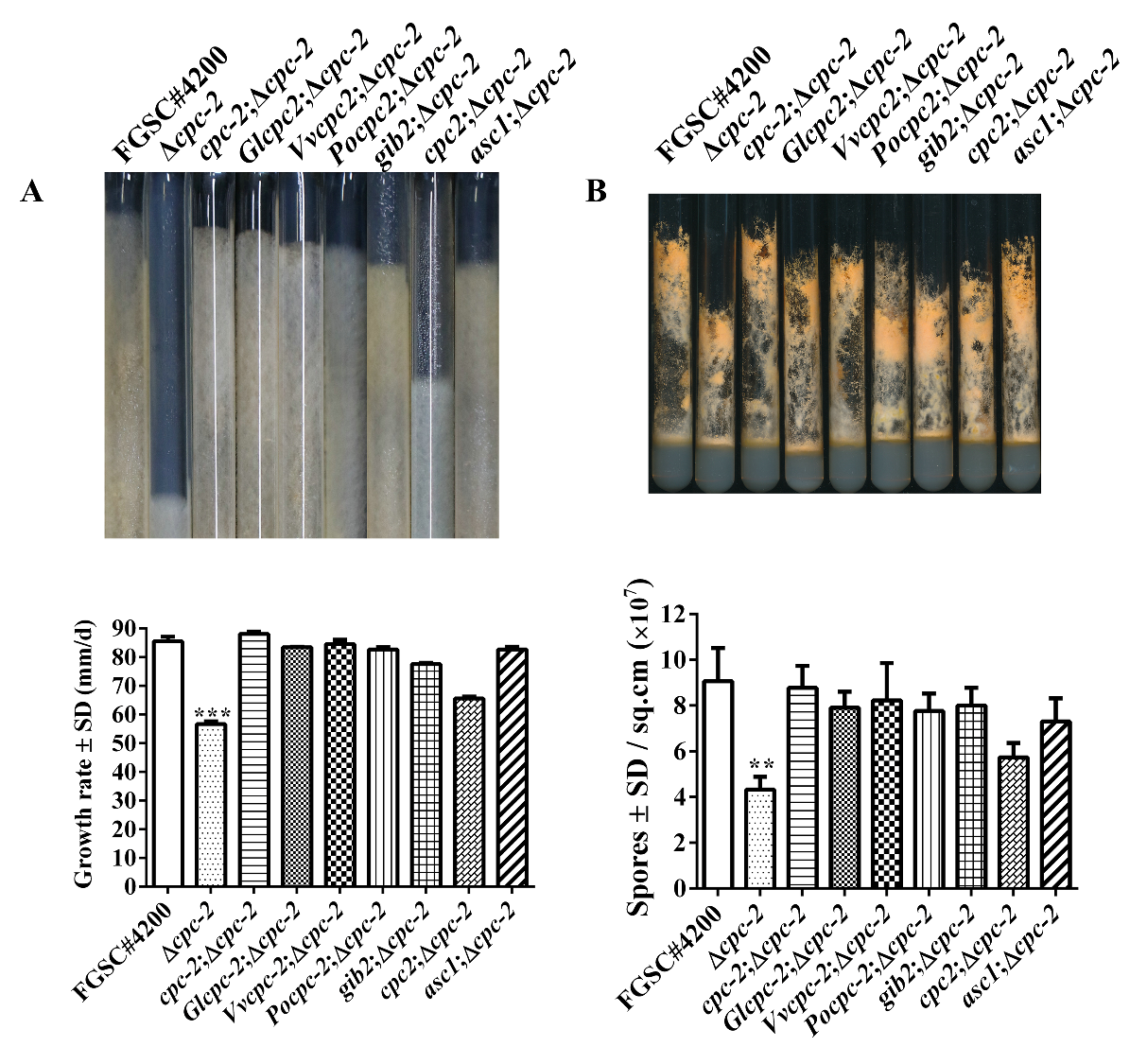
**Figure S2. Complementation of *N. crassa* ∆*cpc-2* with CPC-2 ortholog encoding genes from different fungi in vegetative growth and asexual sporulation.** (**A**) Strains were grown in race tubes and their growth rates were calculated based on 4 days of growth at 28°C. (**B**) Strains were inoculated into test tubes containing Vogel’s agar medium and cultured at 28°C. After 10 days, images of test tubes were captured and conidia produced in each tube were calculated. The strains include wild-type strain FGSC#4200, *cpc-2* deletion mutant (Δ*cpc-2*) and complemented mutants (*cpc-2*;Δ*cpc-2*, *Glcpc2*;Δ*cpc-2*, *Vvcpc2*;Δ*cpc-2*, *Pocpc2*;Δ*cpc-2*, *gib2*;Δ*cpc-2*, *cpc2*;Δ*cpc-2*, *asc1*;Δ*cpc-2*). Values shown are means of three replicates. Standard deviations are indicated with error bars. The significant levels of Δ*cpc-2* in growth rate and conidiation were calculated to wild-type FGSC#4200 by t-test and marked as *** (0.01 < p < 0.05), **** (0.001 < p < 0.01) or ***** (p<0.001).


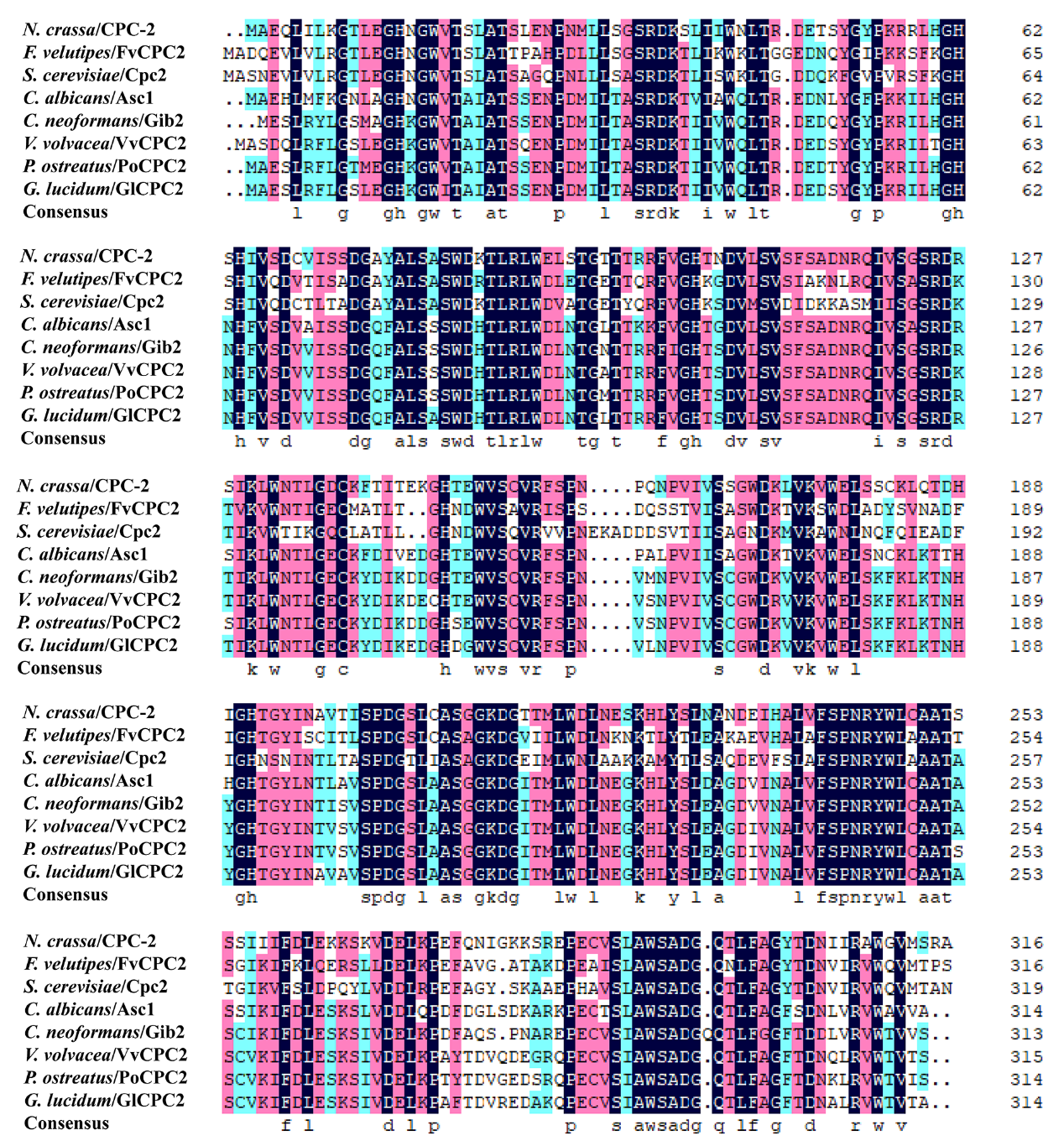
**Figure S3. Sequence alignment analysis of CPC-2 orthologs.** The sequence of CPC-2, FvCPC2, Cpc2, Asc1, Gib2, VvCPC2, PoCPC2 and GlCPC2 from *N. crassa*, *F. velutipes*, *S. cerevisiae*, *C. albicans*, *C. neoformans*, *V. volvacea*, *P.* *ostreatus* and *G.* *lucidum* were obtained from National Center for Biotechnology Information (NCBI, <https://www.ncbi.nlm.nih.gov/gene/>) with the GenBank No. NC_026507.1, KY815023, NM_001182616.1, XM_019475482.1, XP_012053792.1, MN075138, KDQ27929.1, GQ293361.1, respectively. Then the eight sequences were aligned by DNAMAN (Woffelman 2004).


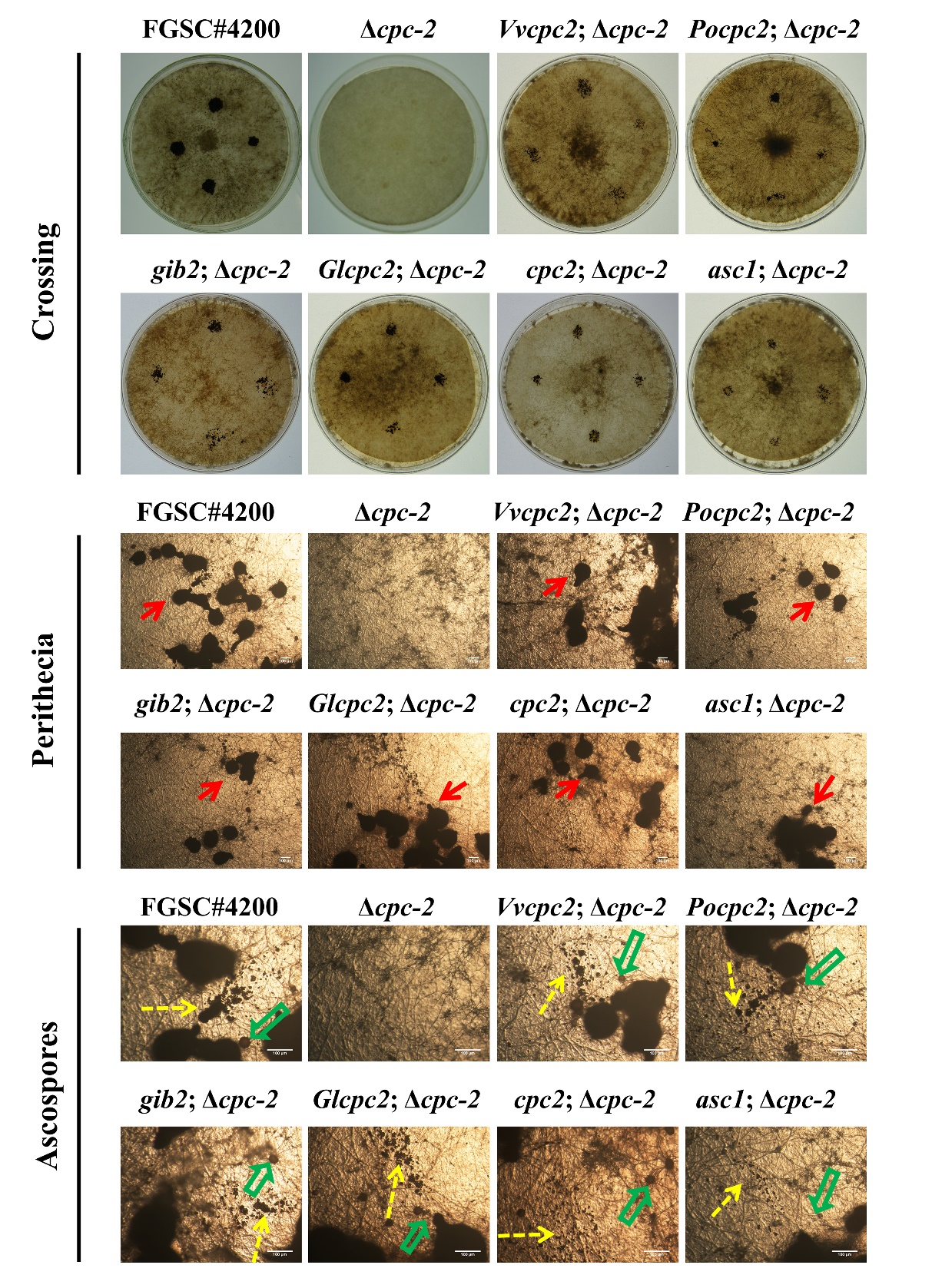
**Figure S4. Complementation of *N. crassa* ∆*cpc-2* with CPC-2 ortholog encoding genes from different fungi in vegetative growth and asexual sporulation.** The wild-type strain FGSC#4200, *cpc-2* deletion mutant (Δ*cpc-2*) and complemented mutants (*Vvcpc2*;Δ*cpc-2*, *Pocpc2*;Δ*cpc-2*, *gib2*;Δ*cpc-2*, *Glcpc2*;Δ*cpc-2*, *cpc2*;Δ*cpc-2*, *asc1*;Δ*cpc-2*; mating type a) were inoculated on SCM plates and incubated at 25°C in the dark. After 7 days, opposite mating type strain of wild type (FGSC#2225, mating type A) were spotted on the colony surface of each strain. Then the plates were cultivated at 25°C under light for another 7 days. The images of protoperithecia and perithecia were captured on the 7th and 14th day by stereomicroscope, respectively. Red arrows indicate perithecia formed. The green blank arrows indicate protoperithecia and the yellow dotted arrows indicate ascospores. The scale bar is 100 μm.

**Table S1. Primers for homokaryon verification and mutant construction in *N. crassa***

| Primer | Sequence (5’ to 3’) | Purpose | Sizes (bp) |
| --- | --- | --- | --- |
| cpc2-ORF-F  cpc2-ORF-R | GCTGGTGGGTGGGCTAAGGA  ATGACACCCCAGGCACGGAT | Amplification of open-reading-frame of *cpc-2* fragment used in homokaryonization | 1469 |
| cpc2-up-F  cpc2-up-R | CAGTTACCCACCAAGATTCC  GGTCAGAAATCGTCAAAGG | Amplification of upstream of *cpc-2* fragment used in homokaryonization | 1278 |
| cpc2-down-F  cpc2-down-R | CTCGGAACTGCTTTGTATCG  ACACTACCTTCACCTTCTCC | Amplification of downstream of *cpc-2* fragment used in homokaryonization | 1193 |
| cpc2-CM-F | AATGGGCCCGACACCAAGGCGAAAGGCAG | Amplification of 5’UTR and full length of *cpc-2* fragment used in Nccpc-2-pCB1532 plasmids | 3459 |
| cpc2-CM-R | AATGGGCCCGTGGTGTGAAGGGAGATTTAGCATA |  |  |
| cpc2-DS-F | GACTAGTCTACACCCTTCTACCCTTCTACCC | Amplification of 3’UTR of *cpc-2* fragment used in Nccpc-2-pCB1532 plasmids | 1551 |
| cpc2-DS-R | ACGCGTCGACTTACAAGACATCAAGACTCCTCGG |  |  |
| NCcpc2-Promoter-F | ACGCGTCGACATACCCAAGGACTTCGAAAACACTT | Amplification of promoter of *cpc-2* used in Fvcpc2-pCB1532 plasmids | 1836 |
| NCcpc2-Promoter-R | CTGAAGCCATCCTTTCTGGTTGATCAGGGGA |  |  |
| Fvcpc2-F | ACCAGAAAGGATGGCTTCAGACCAATTGCG | Amplification of ORF of *Fvcpc2* fragment used in Fvcpc2-pCB1532 plasmids | 948 |
| Fvcpc2-R | TCCGACGTCTTTATGAGGTGACAGTCCAGACACG |  |  |
| NCcpc2-Terminator-F | CACCTCATAAAGACGTCGGAGCCGGGACT | Amplification of terminator of *cpc-2* used in Fvcpc2-pCB1532 plasmids | 853 |
| NCcpc2-Terminator-R | GGGGTACCGAGGACGACTTGAGTGAGCGG |  |  |
| Promoter-cpc2-F | GTCTTGTAAGTCGACATACCCAAGGACTTCGAAAACACTT | Amplification of promoter of *cpc-2* used in Sccpc2-pCB1532 plasmids | 1738 |
| PromoterSCcpc2-R | TAGATGCCATCCTTTCTGGTTGATCAGGGGA |  |  |
| SCcpc2-F | ACCAGAAAGGATGGCATCTAACGAAGTTTTAGTTT | Amplification of ORF of *cpc2* fragment used in Sccpc2-pCB1532 plasmids | 960 |
| SCcpc2-R | TCCGACGTCTTTAGTTAGCAGTCATAACTTGCCAA |  |  |
| Terminator-SCcpc2-F | TGCTAACTAAAGACGTCGGAGCCGGGACT | Amplification of terminator of *cpc-2* used in Sccpc2-pCB1532 plasmids | 539 |
| Terminator-cpc2-R | GGCGAATTGGGTACCGAGGACGACTTGAGTGAGCGG |  |  |
| Promoter-cpc2-F | GTCTTGTAAGTCGACATACCCAAGGACTTCGAAAACACTT | Amplification of promoter of *cpc-2* used in Caasc1-pCB1532 plasmids | 1738 |
| PromoterCAcpc2-R | GATCAGCCATCCTTTCTGGTTGATCAGGGGA |  |  |
| CAasc1-F | ACCAGAAAGGATGGCTGATCAAGAAGTTTTAGTTT | Amplification of ORF of *asc1* fragment used in Caasc1-pCB1532 plasmids | 954 |
| CAasc1-R | TCCGACGTCTTTAAGCAGATGGAGTCATAACTTGC |  |  |
| Terminator-CAasc1-F | ATCTGCTTAAAGACGTCGGAGCCGGGACT | Amplification of terminator of *cpc-2* used in Caasc1-pCB1532 plasmids | 539 |
| Terminator-cpc2-R | GGCGAATTGGGTACCGAGGACGACTTGAGTGAGCGG |  |  |
| Promoter-cpc2-F | GTCTTGTAAGTCGACATACCCAAGGACTTCGAAAACACTT | Amplification of promoter of *cpc-2* used in Glcpc2-pCB1532 plasmids | 1738 |
| Promoter-GLcpc2-R | GAGATTCCATCCTTTCTGGTTGATCAGGGGA |  |  |
| GLcpc2-F | ACCAGAAAGGATGGAATCTCTCCGCTACCTCG | Amplification of ORF of *Glcpc2* fragment used in Glcpc2-pCB1532 plasmids | 942 |
| GLcpc2-R | TCCGACGTCTTTATGAGACGACAGTCCACACCC |  |  |
| Terminator-GLcpc2-F | CGTCTCATAAAGACGTCGGAGCCGGGACT | Amplification of terminator of *cpc-2* used in Glcpc2-complemention plasmids | 539 |
| Terminator-cpc2-R | GGCGAATTGGGTACCGAGGACGACTTGAGTGAGCGG |  |  |
| Promoter-cpc2-F | GTCTTGTAAGTCGACATACCCAAGGACTTCGAAAACACTT | Amplification of promoter of *cpc-2* used in Cngib2-pCB1532 plasmids | 1738 |
| CNgib2-P-R | GCTCGGCCATCCTTTCTGGTTGATCAGGGGA |  |  |
| CNgib2-F | ACCAGAAAGGATGGCCGAGCACCTCATGT | Amplification of ORF of *gib2* fragment used in Cngib2-pCB1532 plasmids | 945 |
| CNgib2-R | TCCGACGTCTCTAAGCAACGACAGCCCAGACT |  |  |
| CNgib2-T-F | CGTTGCTTAGAGACGTCGGAGCCGGGACT | Amplification of terminator of *cpc-2* used in Cngib2-pCB1532 plasmids | 539 |
| Terminator-cpc2-R | GGCGAATTGGGTACCGAGGACGACTTGAGTGAGCGG |  |  |
| Promoter-cpc2-F | GTCTTGTAAGTCGACATACCCAAGGACTTCGAAAACACTT | Amplification of promoter of *cpc-2* used in Vvcpc2-pCB1532 plasmids | 1738 |
| Promoter-VVcpc2-R | ATTCTGCCATCCTTTCTGGTTGATCAGGGGA |  |  |
| VVcpc2-F | ACCAGAAAGGATGGCAGAATCACTGCGTTTC | Amplification of ORF of *Vvcpc2* fragment used in Vvcpc2-pCB1532 plasmids | 945 |
| VVcpc2-R | TCCGACGTCTTTATGCTGTAACGGTCCAGACTC |  |  |
| Terminator-VVcpc2-F | TACAGCATAAAGACGTCGGAGCCGGGACT | Amplification of terminator of *cpc-2* used in Vvcpc2-pCB1532 plasmids | 539 |
| Terminator-cpc2-R | GGCGAATTGGGTACCGAGGACGACTTGAGTGAGCGG |  |  |
| Promoter-cpc2-F | GTCTTGTAAGTCGACATACCCAAGGACTTCGAAAACACTT | Amplification of promoter of *cpc-2* used in Pocpc2-pCB1532 plasmids | 1738 |
| Promoter-POcpc2-R | ATTCCGCCATCCTTTCTGGTTGATCAGGGGA |  |  |
| POcpc2-F | ACCAGAAAGGATGGCGGAATCTTTACGCTTT | Amplification of ORF of *Pocpc2* fragment used in Pocpc2-pCB1532 plasmids | 942 |
| POcpc2-R | TCCGACGTCTTGAGATCACAGTCCAGACCCG |  |  |
| POcpc2-T-F | TGTGATCTCAAGACGTCGGAGCCGGGACT | Amplification of terminator of *cpc-2* used in Pocpc2-pCB1532 plasmids | 539 |
| Terminator-cpc2-R | GGCGAATTGGGTACCGAGGACGACTTGAGTGAGCGG |  |  |
| cpc2-detect-F | ATACCCAAGGACTTCGAAAACACTT | Verification the complemented plasmids | 3219 |
| cpc2-detect-R | GAGGACGACTTGAGTGAGCGG |  |  |

**Table S2. Primers for mutant construction in *F. velutipes***

| Primer | Sequence (5’ to 3’) | Purpose |
| --- | --- | --- |
| XmnI-Pgpd-F | CAGATCCCCCGAATTATTCGAGCTCGGTACAGTCGTG | Amplification of P*gpd* fragment used in *Fvcpc2* Overexpression plasmids |
| Pgpd-Fvcpc2OE-R | TCCAGCCACCGACCTGTAAAATGGTGAGCAAGAC |  |
| Fvcpc2OE-Pgpd-F | TTTACAGGTCGGTGGCTGGAAGCGTGACATC | Amplification of *Fvcpc2* fragment used in *Fvcpc2* Overexpression plasmids |
| Fvcpc2OE-Pgpd-R | AAGTGGATCCTTATGAGGTGACAGTCCAGACACG |  |
| TtrpC-Fvcpc2OE-F | CACCTCATAAGGATCCACTTAACGTTACTGAAATCA | Amplification of T*trpC* fragment used in *Fvcpc2* Overexpression plasmids |
| XmnI-TtrpC-R | AATTAACGCCGAATTCATGCCTGCAGGTCGAGAAAG |  |
| Pgpd-Fvcpc2-F | TACGAATTCGAGCTCGGTACCAGTCGTGGGTCCAGCATTTTG | Amplification of P*gpd* fragment used in *Fvcpc2* RNAi plasmids |
| Pgpd-Fvcpc2-R | GATGAGTGTCACACCGAATGGACCTGTAAAATGGTGAGCAAGAC |  |
| Fvcpc2-antisense-F | ACCATTTTACAGGTCCATTCGGTGTGACACTCATCTTTG | Amplification of *Fvcpc2 anti*sense fragment for the construction of pre-Fvcpc2-RNAi |
| Fvcpc2-antisense-R | TCGGCGCGATGATACACAAAACTATCATTGTGTGGCAGC |  |
| Fvcpc2-sense-F | CAATGATAGTTTTGTGTATCATCGCGCCGACTTTTC | Amplification of *Fvcpc2* sense fragment linked with the spacer for the construction of Fvcpc2-RNAi |
| Fvcpc2-sense-R | ACGTTAAGTGGATCCCATTCGGTGTGACACTCATCTTTG |  |
| TtrpC-Fvcpc2RNAi-F | GTGTCACACCGAATGGGATCCACTTAACGTTACTGAAATCAT | Amplification of T*trpC* fragment used in *Fvcpc2* RNAi plasmids |
| TtrpC-Fvcpc2RNAi-R | TTGCATGCCTGCAGGTCGACGAAAGAAGGATTACCTCTAAACAAGTGT |  |
| Pgpd-detect-F | AACCGCCATCTTCCACACTT | Verification the two entire constructions: P*gpd*-Fvcpc2OE-T*trpC* and P*gpd*-Fvcpc2RNAi-T*trpC* |
| TtrpC-detect-R | AACACCATTTGTCTCAACTCCG |  |

**Table S3. Primers for qPCR**

| Primer | Sequence (5’ to 3’) | Targets | Sizes of the target (bp) |
| --- | --- | --- | --- |
| Q-FvActin-F | CACCATGTTCCCTGGTATTG | *β-actin* of  *F. velutipes* | 106 bp |
| Q-FvActin-R | CACCAATCCAGACAGAGTATTT |  |  |
| Q-Fvcpc2-F | CGATACTGGCTCTGTGCAGCTA | *Fvcpc2* of  *F. velutipes* | 126 bp |
| Q-Fvcpc2-R | ACACTCGGGTTGCCTTCCTT |  |  |
| Q-gene10451-F | CGGTTATTGCAGACGGGTTAT | gene10451 of  *F. velutipes* | 101 bp |
| Q-gene10451-R | AGTGCCTCGCTCTTGAATATG |  |  |
| Q-gene8023-F | CCAAGGAGTGCTCAATAAC | gene8023 of  *F. velutipes* | 132 bp |
| Q-gene8023-R | CGAGCATCTCGTAGATCA |  |  |
| Q-Fv-JRL1-F | CTGACAAAGGCAGAGTAAC | *Fv-JRL1* of  *F. velutipes* | 142 bp |
| Q-Fv-JRL1-R | CCTTGACGATGGAGATAGA |  |  |
| Q-gene10415-F | TGTCATCAAAGAGTCGGATA | gene10145 of  *F. velutipes* | 129 bp |
| Q-gene10415-R | TTGACGAGGGAGAGAGA |  |  |
| Q-gene10856-F | AGGGCTGTCATCAAAGAG | gene10856 of  *F. velutipes* | 129 bp |
| Q-gene10856-R | GAGAGAGAGAGAGGTGAGA |  |  |
| Q-gene9094-F | CTCGCTAAAGTCGGTAGTA | gene9094 of  *F. velutipes* | 107 bp |
| Q-gene9094-R | CCGATGTTGGTGGAATTG |  |  |
| Q-FVFD16-F | AGGTTGCTGCTGTTAGT | *FVFD16* of  *F. velutipes* | 103 bp |
| Q-FVFD16-R | AACAGGAGGAGTGTGATG |  |  |
| Q-gene543-F | GACATCGTCAAGAACATCAA | gene543 of  *F. velutipes* | 122 bp |
| Q-gene543-R | AATCCCGGAACCAAGAA |  |  |
| Q-FVFD30-F | CAGAAGCAAGCAGGAAAG | *FVFD30* of  *F. velutipes* | 150 bp |
| Q-FVFD30-R | GTAACCAACTCCGTCATTC |  |  |
| Q-Hyd-1-F | GGTCTTGTTGGTTTGACTT | *Hyd-1* of  *F. velutipes* | 123 bp |
| Q-Hyd-1-R | GGAGCATCCCAAATTAATGA |  |  |
| Q-Hyd-3-F | CATCGGTGGATTGAACAG | *Hyd-3* of  *F. velutipes* | 102 bp |
| Q-Hyd-3-R | CAGAGGCTGATTGGAATG |  |  |
| Q-Hyd-4-F | GCTCATCTTGAACCTCCT | *Hyd-4* of  *F. velutipes* | 120 bp |
| Q-Hyd-4-R | TTGTCCGTGCAACAAAC |  |  |
| Q-Hyd-6-F | GCACTCTTGGGTCTTCT | *Hyd-6* of  *F. velutipes* | 132 bp |
| Q-Hyd-6-R | CCCAAAGCTGTTGTTCTC |  |  |
| Nc-β-tublin-F | CCCAAGAACATGATGGCTGCTTCT | *β-tublin* of  *N. crassa* | 122 bp |
| Nc-β-tublin-R | TTGTTCTGAACGTTGCGCATCTGG |  |  |
| Q-cr-1-F | GTCAACAAGGCTAGTCGTATC | *cr-1* of  *N. crassa* | 118 bp |
| Q-cr-1-R | CAACATCAACCGACTCCTTAT |  |  |
| Q-pkac-2-F | CATCAAGCTGGTCGACTTT | *pkac-2* of  *N. crassa* | 136 bp |
| Q-pkac-2-R | TGTCGTGTGGCCTTTATTC |  |  |
| Q-poi-2-F | ATCGACAAGCGTCACAAC | *poi-2* of  *N. crassa* | 145 bp |
| Q-poi-2-R | CGACAGAACCACGCTTATC |  |  |

**Table S4. The time line of basidioma development in wild type (F19) and *Fvcpc2* mutants**

| **Strains** | **Time for complete spawn run** | | **Time for primordia appearance** | **Time for harvest** |
| --- | --- | --- | --- | --- |
|  | ***^a^*R1 R2 R3** | | **R1 R2 R3** | **R1 R2 R3** |
| F19 | | 13 days 16 days 15 days | 28 days 30 days 31 days | 41 days 43 days 42 days |
| *Fvcpc2*OE#5 | | 13 days 16 days 15 days | 27 days 29 days 30 days | 38 days 40 days 39 days |
| *Fvcpc2*OE#33 | | 13 days 16 days 15 days | 27 days 29 days 30 days | 38 days 40 days 39 days |
| *Fvcpc2*OE#124 | | 13 days 16 days 15 days | 27 days 29 days 30 days | 38 days 40 days 39 days |

*^a^*The fruiting body experiments were taken three biological repeats (R1, R2 a R3), and every repeat contains six bottles.
